# Engineered CD4 T cells expressing a membrane anchored viral inhibitor restrict HIV-1 through *cis* and *trans* mechanisms

**DOI:** 10.3389/fimmu.2023.1167965

**Published:** 2023-09-14

**Authors:** Weiming Wang, Khanghy Truong, Chaobaihui Ye, Suman Sharma, Huan He, Lihong Liu, Michael Wen, Anisha Misra, Paul Zhou, Jason T. Kimata

**Affiliations:** ^1^ Unit of Anti-Viral Immunity and Genetic Therapy, Key Laboratory of Molecular Virology and Immunology, Institute Pasteur of Shanghai, Chinese Academy of Sciences, Shanghai, China; ^2^ Department of Molecular Virology and Microbiology, Baylor College of Medicine, Houston, TX, United States

**Keywords:** HIV, CD4 + T cells, dendritic cells, neutralization, single chain variable fragments, glycosyl phosphatidylinositol, immunotherapy

## Abstract

HIV-1 infection of target cells can occur through either cell-free virions or cell-cell transmission in a virological synapse, with the latter mechanism of infection reported to be 100- to 1,000-fold more efficient. Neutralizing antibodies and entry inhibitors effectively block cell-free HIV-1, but with few exceptions, they display much less inhibitory activity against cell-mediated HIV-1 transmission. Previously, we showed that engineering HIV-1 target cells by genetically linking single-chain variable fragments (scFvs) of antibodies to glycosyl phosphatidylinositol (GPI) potently blocks infection by cell-free virions and cell-mediated infection by immature dendritic cell (iDC)-captured HIV-1. Expression of scFvs on CD4^+^ cell lines by transduction with X5 derived anti-HIV-1 Env antibody linked to a GPI attachment signal directs GPI-anchored scFvs into lipid rafts of the plasma membrane. In this study, we further characterize the effect of GPI-scFv X5 on cell-cell HIV-1 transmission from DCs to target cells. We report that expression of GPI-scFv X5 in transduced CD4^+^ cell lines and human primary CD4^+^ T cells potently restricts viral replication in iDC- or mDC-captured HIV-1 *in trans*. Using live-cell imaging, we observed that when GPI-GFP or GPI-scFv X5 transduced T cells are co-cultured with iDCs, GPI-anchored proteins enrich in contact zones and subsequently migrate from T cells into DCs, suggesting that transferred GPI-scFv X5 interferes with HIV-1 infection of iDCs. We conclude that GPI-scFv X5 on the surface of transduced CD4^+^ T cells not only potently blocks cell-mediated infection by DCs, but it transfers from transduced cells to the surface of iDCs and neutralizes HIV-1 replication in iDCs. Our findings have important implications for HIV-1 antibody-based immunotherapies as they demonstrate a viral inhibitory effect that extends beyond the transduced CD4+ T cells to iDCs which can enhance HIV-1 replication.

## Introduction

Upon crossing the epithelial barrier, HIV-1 encounters and infects a wide range of cells in the underlying mucosal tissues including professional antigen presenting cells such as dendritic cells through CCR5 mediated viral entry. Immature DCs (iDCs) play a significant role in promoting the dissemination and amplification of HIV-1 infection. iDCs capture HIV-1 and facilitate infection of nearby CD4^+^ T cells by trafficking captured virions to draining lymphoid tissues where, upon transition to mature DCs (mDCs), they transmit the virus to CD4^+^ T cells (1–3). *In vitro* the transmission of HIV-1 from DCs to CD4^+^ T cells occurs via two mechanisms: 1) *trans*-infection, i.e. cell-cell transmission by capture and transfer of HIV-1 from iDCs or mDCs to CD4^+^ T cells which can occur within a few hours post infection with or without virus internalization (4–9); and 2) *cis*-infection, i.e. cell-free transmission where nascent virions from infected iDCs bud and infect CD4^+^ T cells which can occur within a few days and is sensitive to antiviral therapy (ART) (8–12).

iDCs or mDCs can mediate cell-cell transmission of captured HIV-1 to CD4^+^ T cells via virological synapses (13). Within the viral synapse, captured virions are liberated from their invaginated holding membrane compartment of DCs and engage CD4 and co-receptors (CCR5 or CXCR4) on CD4^+^ T cells. This high local concentration of virions enhances the efficiency of HIV-1 transmission (6, 7). Indeed, cell-cell *trans*-infection appears to be 100- to 1,000-fold more efficient for the spread of HIV-1 *in vitro* (7, 14, 15). While the relative impact of cell-free and cell-cell transmission *in vivo* remains to be defined, in a bone marrow-liver-thymus (BLT) humanized mouse model, HIV-1-infected CD4^+^ T cells in lymph nodes were found to be mobile, capable of establishing virological synapses, and form syncytia. Of note, blocking egress of migratory T cells from lymph nodes into efferent lymph vessels and interrupting T cell recirculation by the use of a S1PR1 (sphingosine 1-phosphate receptor 1) antagonist FTY720 at early onset of HIV-1 infection resulted in limited HIV-1 dissemination and reduction of plasma viremia (16), indicating that cell-cell transmission of HIV-1 may be important in establishment of systemic HIV-1 infection.

Neutralizing antibodies and pharmacologic entry inhibitors effectively block cell-free transmission of HIV-1. However, with few exceptions, they are less capable of blocking cell-cell viral transmission (13–15, 17–26). In T cell-T cell co-culture where HIV-infected donor T cells were added to uninfected T cells, viral neutralization was achieved only when virus-infected donor T cells were pretreated with antibodies before being added to target T cells (14, 15, 18, 19, 24–26). Additionally, Reh et al. tested a panel of 16 broadly neutralizing antibodies against 11 HIV-1 strains using cell-free virus and cell-cell viral transmission assays and concluded that capacity of broadly neutralizing antibodies to inhibit cell-cell viral transmission of HIV-1 is not only strain- and epitope-dependent, but also dependent on the window of action during the entry process in early infection (19). Further, the reports on *trans*-infection of DC-CD4^+^ T cell in co-cultures have been variable due to variations in assay systems used by different research groups (17, 20–23, 27, 28). For example, Su et al. showed that targeting HIV-1 entry receptor gp120 with antibodies 2G12, b12, VRC01, VRC03 and targeting HIV-1 fusion receptor gp41 with antibodies 2F5 and 4E10 blocked HIV-1 *trans*-infection (17). Conversely, Sagar et al. showed that only anti-gp41 antibodies 2F5 and 4E10, but not anti-gp120 antibodies 2G12, b12, VRC01, and PG16 could block *trans*-infection *(* 23). Moreover, van Montfort et al. showed that HIV-1 bound to antibodies 2F5, 4E10 and 10E8, but not bound to b12, NIH45-46, and VRC01, could still be captured by DCs and subsequently infect CD4^+^ T cells (21, 22).

Lipid rafts in the plasma membrane are critical for the formation of virological synapses that mediate cell-cell transmission (29). Lipid rafts are specialized dynamic micro-domains of plasma membrane enriched for cholesterol, sphingolipids, and glycerophospholipids (30). They have been shown to function as doorways of HIV-1 budding as well as for HIV-1 entry into CD4^+^ T cells and macrophages (31–34). Importantly, the CD4 receptor has been found to be located within lipid rafts of plasma membrane (35, 36). Thus, directing HIV-1 neutralizing antibodies to lipid rafts within the virological synapse would be expected to enhance restriction of cell-cell viral transmission.

Previously, we showed that engineering CD4^+^ T cells to express membrane anchored viral inhibitors (MAVIs) protects modified cells from depletion and potently restricts HIV-1 (37). We genetically linked single chain variable fragments (scFv) or heavy chain third complementarity determining region (HCDR3) of human monoclonal antibodies specific for the HIV-1 envelope with a glycosyl phosphatidylinositol (GPI) attachment signal derived from decay accelerating factor (DAF) (38). Using these constructs, we demonstrated that the GPI anchor targeted the scFvs or HCDR3 molecules to lipid rafts of the plasma membrane of cells. While inhibition primarily occurred by blocking virus-receptor interactions, we also showed that GPI-anchored scFvs targeting the HIV Env can block viral replication from within cells, inhibiting Env incorporation into virions and reducing viral infectivity (39). Interestingly, GPI-scFvs based on antibodies X5 and 48d as well as GPI-HCDR3 PG9 and PG16 exhibited potent and broad neutralizing activity against diverse cell-free and iDC-captured HIV-1 infection (37, 40). In this study, we investigated the effect of GPI-scFv X5 expression on CD4^+^ cell lines and primary human CD4^+^ T cells with regards to their ability to block trans-infection of HIV-1 captured by iDCs or mDCs. Utilizing virological and live-cell imaging methods, we found that GPI-scFv X5 not only significantly inhibits cell-mediated infection by DCs, but also transfers to the surface of iDCs, leading to neutralization of HIV-1 replication within these cells. Taken together, our results suggest that using GPI-scFv-based MAVIs that can effectively restrict both HIV-1 cis- and trans-infection may be a promising approach to achieve a functional cure for HIV.

## Results

### DEAE-dextran increases cell-free HIV-1 infection, but not DC-mediated trans-infection

**Figure 1:**
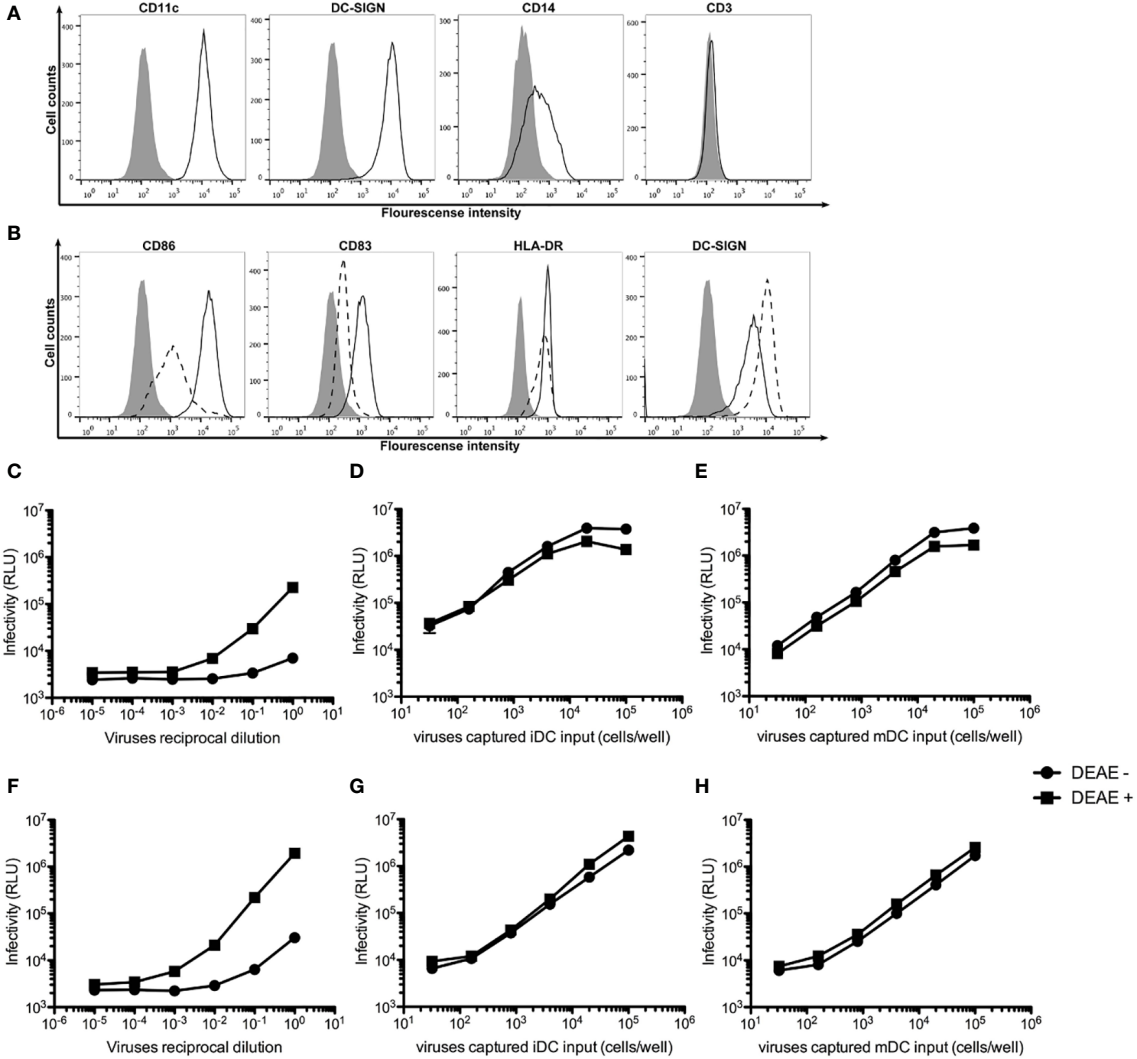
*DEAE-dextran increases cell-free HIV-1 infection, but not trans-infection of HIV*-1. **(A)** Histogram analysis of the expression of CD11c, CD209 (DC-SIGN), CD14 and CD3 on the surface of human monocyte-derived iDCs (open area) as compared unstained cells (solid area). **(B)** Histogram analysis of the expression of the maturation markers CD86, CD83, CD209 and HLA-DR on the surface of mDCs (continuous lines) as compared with iDCs (dash line). **(C, D)** Infectivity as measured by Relative Light Units (RLU) to TZM.bl cells by various reciprocal dilutions of HIV-1 AD8 **(C)** or Bru-3 **(D)** stocks in the presence or absence of DEAE-Dextran. **(E, F)** Infectivity as measured by RLU to TZM.bl cells by various amount of HIV-1 AD8 **(E)** or Bru-3 **(F)** captured by iDCs in the presence or absence of DEAE-Dextran. **(G, H)** Infectivity as measured by RLU to TZM.bl cells by various amount of HIV-1 AD8 **(G)** or Bru-3 **(H)** captured by mDCs in the presence or absence of DEAE-Dextran.

To generate monocyte-derived iDCs and mDCs, human monocytes were isolated from peripheral blood mononuclear cells (PBMCs) using anti-CD14 antibody-coated beads and stimulated with GM-CSF and IL-4 as previously described (37). **Figure 1A** shows that human monocyte-derived iDCs expressed high levels of CD209 (DC-SIGN) and CD11c, reduced level of CD14, but not CD3, indicating the absence of contaminating T cells in the cultures of iDCs. The iDCs were then maturated into mDCs with LPS stimulation. **Figure 1B** shows that mDCs expressed higher levels of CD86, CD83 and HLA-DR, but lower levels of CD209, as compared to those in iDCs.

DEAE-dextran has been shown to significantly increase cell-free HIV-1 infection, but to have little effect on cell-cell transmission of HIV-1 (14). Therefore, we tested the effect of DEAE-dextran on cell-free HIV-1 infection versus iDC- or mDC-mediated *trans*-infection of HIV-1. **Figures 1C–H** show that indeed, DEAE-dextran significantly increased cell-free HIV-1 infection (C and D), but has little effect on iDC- or mDC-mediated *trans*-infection (E to H). In the absence of DEAE-dextran, iDC- or mDC-mediated *trans*-infection was at least 100-fold more effective than free HIV-1 infection. Thus, all our subsequent DC-mediated *trans*-infection experiments were carried out in the absence of DEAE-dextran so that under this condition, the contribution of free HIV-1 infection is negligible.

### GPI-scFv X5-transduced CD4^+^ cell lines resist trans-infection by iDC- or mDC-captured HIV-1

**Figure 2:**
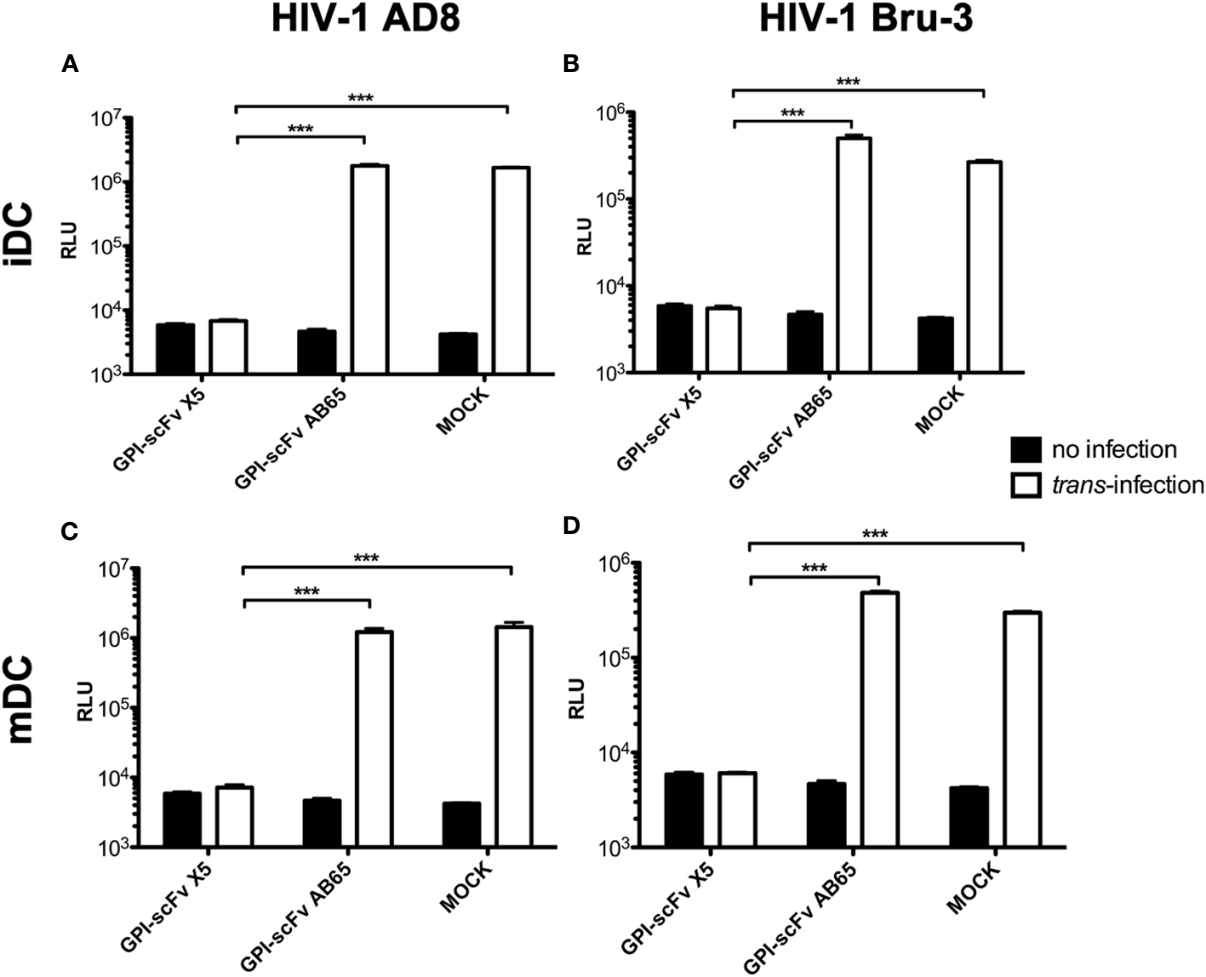
*GPI-scFv X5-transduced TZM.bl cells are resistant to iDC- or mDC-mediated trans-infection of HIV-1.* **(A)** RLU in mock-, GPI-scFv X5 or AB65-transduced TZM.bl cells with or without *trans*-infection of iDC-captured HIV-1 AD8. *** stands for *P* values = or < 0.001. **(B)** RLU in mock-, GPI-scFv X5 or AB65-transduced TZM.bl cells with or without *trans*-infection of iDC-captured HIV-1 Bru-3. *** stands for *P* values = or < 0.001. **(C)** RLU in mock-, GPI-scFv X5 or AB65-transduced TZM.bl cells with or without *trans*-infection of mDC-captured HIV-1 AD8. Infections were performed in triplicate. The average values ± standard deviation (SD) are shown. *** stands for *P* values = or < 0.001. **(D)** RLU in mock-, GPI-scFv X5 or AB65-transduced TZM.bl cells with or without *trans*-infection of mDC-captured HIV-1 Bru-3. *** stands for *P* values = or < 0.001.

To test the effect of GPI-scFv X5 on *trans*-infection by iDC-captured HIV-1, iDCs from healthy donors were incubated with CCR5-tropic HIV-1 AD8 or CXCR4-tropic HIV-1 Bru-3 for 2 hours. After the infection, cells were washed extensively to remove cell-free virions. TZM.bl reporter cells were used to evaluate *trans*-infection by iDC-captured HIV-1. iDCs with or without captured HIV-1 were co-cultured with TZM.bl, TZM.bl expressing GPI-scFv X5 or the GPI-scFv AB65 negative control construct. **Figure 2** shows that while TZM.bl, TZM.bl-GPI-scFv X5 or AB65 cells alone yielded background levels of Relative Light Units (RLU), TZM.bl and TZM.bl-GPI-scFv AB65 cells co-cultured with iDCs harboring captured HIV-1 AD8 or Bru-3 exhibited significantly increased levels of RLU. In contrast, TZM.bl-GPI-scFv X5 cells co-cultured with iDCs containing captured HIV-1 AD8 or Bru-3 exhibited close to background levels of RLU (**Figures 2A, B**), indicating that in transduced TZM.bl cells GPI-scFv X5 significantly blocked *trans*-infection of HIV-1 by iDCs. Similar results were observed when mDCs with or without captured HIV-1 AD8 or Bru-3 were co-cultured with TZM.bl, TZM.bl-GPI-scFv X5 or AB65 cells (**Figures 2C, D**). These results have been observed in two other experiments using iDCs and mDCs derived from additional donors (**Figure S1**).

**Figure 3:**
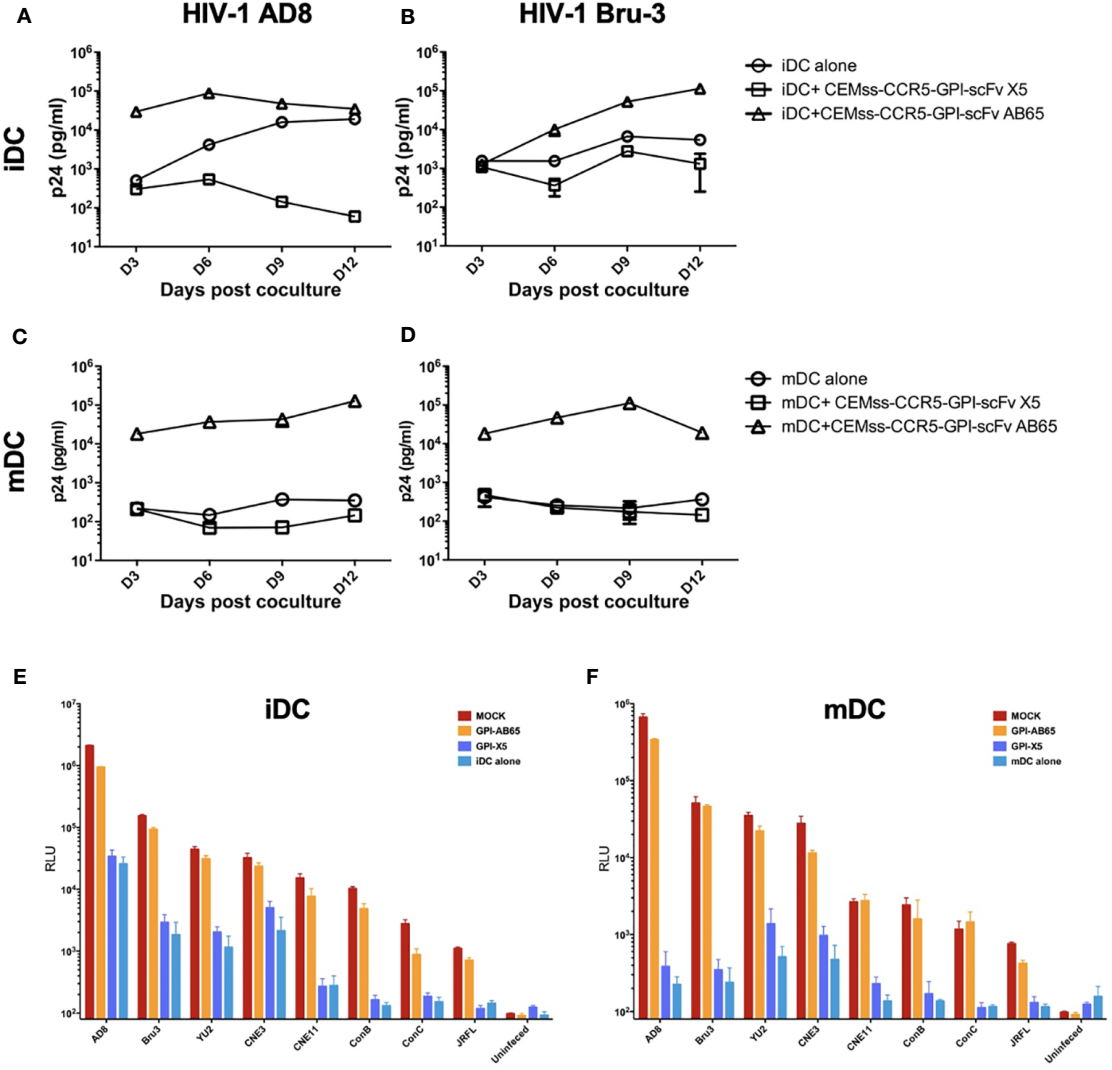
*GPI-scFv X5-transduced CEMss-CCR5 cells are resistant to iDC- or mDC-mediated trans-infection of HIV-1.* **(A)** HIV-1 gag p24 in the culture supernatants collected at days 3, 6, 9 and 12 post co-culture between mock-, GPI-scFv X5 or AB65-transduced CEMss-CCR5 cells and HIV-1 AD8-captured iDCs. **(B)** HIV-1 gag p24 in the culture supernatants collected at days 3, 6, 9 and 12 post co-culture between mock-, GPI-scFv X5 or AB65-transduced CEMss-CCR5 cells and HIV-1 Bru-3-captured iDCs. **(C)** HIV-1 gag p24 in the culture supernatants collected at days 3, 6, 9 and 12 post co-culture between mock-, GPI-scFv X5 or AB65-transduced CEMss-CCR5 cells and HIV-1 AD8-captured mDCs. **(D)** HIV-1 gag p24 in the culture supernatants collected at days 3, 6, 9 and 12 post co-culture between mock-, GPI-scFv X5 or AB65-transduced CEMss-CCR5 cells and HIV-1 Bru-3-captured mDCs. **(E)** RLU detected in mock-, GPI-scFv X5 or AB65-transduced CEMss-CCR5 cells *trans*-infected with or without HIV-1 pseudotyped AD8, Bru-3, Yu2, CNE3, CNE11, Con B, Con C or JRFL-captured iDCs at 48 hours post co-culture. RLU in HIV-1 pseudotyped AD8, Bru-3, Yu2, CNE3, CNE11, Con B, Con C or JRFL-captured iDCs alone were included for the comparison. **(F)** RLU detected in mock-, GPI-scFv X5 or AB65-transduced CEMss-CCR5 cells *trans*-infected with or without HIV-1 pseudotyped AD8, Bru-3, Yu2, CNE3, CNE11, Con B, Con C or JRFL-captured mDCs at 48 hours post co-culture. RLU in HIV-1 pseudotyped AD8, Bru-3, Yu2, CNE3, CNE11, Con B, Con C or JRFL-captured mDCs alone were included for the comparison. Infections were performed in triplicate. The average values ± standard deviation (SD) are shown.

While TZM.bl cells are a powerful tool for evaluating HIV-1 infection, they are of epithelial origin. We next tested the effect of GPI-X5 on *trans*-infection using CEMss T cells modified to express CCR5 as target cells. We found that depending on the coreceptor usage of the captured virus, distinctly different results were observed with iDCs. Interestingly, co-culture of the iDCs with captured R5-tropic HIV-1 AD8 and CEMss-CCR5-GPI-scFv X5 resulted in decreasing virus production compared to iDCs with HIV-1 AD8 alone, which increased over time. High levels of HIV-1 production were observed in co-cultures of iDCs with captured HIV-1 AD8 and CEMss-CCR5-GPI-scFv AB65 (**Figure 3A**). Co-culture of iDCs with captured X4-tropic HIV-1 Bru-3 and CEMss-CCR5-GPI-scFv X5 resulted in a constant but very low level of HIV-1 production similar to iDCs with captured HIV-1 Bru-3 alone, suggesting limited infection and spread. In contrast, co-culture of iDCs with captured HIV-1 Bru-3 and CEMss-CCR5-GPI-scFv AB65 resulted in a significantly increased level of HIV-1 replication (**Figure 3B**).

When mDCs were used to mediate transmission of HIV-1, similar results were observed with R5- and X4-tropic viruses. Co-cultures of mDCs with captured HIV-1 AD8 or Bru-3 and CEMss-CCR5-GPI-scFv X5 resulted in very low level of HIV-1 replication like mDCs with HIV-1 AD8 or Bru-3. In contrast, co-culture of mDCs with captured HIV-1 AD8 or Bru-3 and CEMss-CCR5 expressing the negative control GPI-scFv AB65 construct resulted in a significantly higher level of HIV-1 production (**Figures 3C, D**). Similar results were observed in two additional experiments using iDCs and mDCs derived from different donors (**Figure S2**).

To specifically test the initial blocking of *trans*-infection by GPI-scFv X5, we performed single round infections with a panel of iDC- or mDC-captured HIV-luc pseudotyped with different Env proteins. **Figures 3E, F** show that inhibition of *trans*-infection by GPI-scFv X5 occurred when CEMss-CCR5-GPI-scFv X5 cells, but not CEMs-CCR5 or CEMss-CCR5-GPI-scFv AB65 cells, were co-cultured with iDCs or mDCs containing captured HIV-1 virions. These results were confirmed in two other independent experiments using iDCs and mDCs derived different donors (**Figure S3**).

Taken together, these data indicate that GPI-scFv X5 significantly blocks iDC or mDC-mediated *trans*-infection of CD4+ target cells by HIV-1. These results verify and extend our previously findings with iDCs (37). Furthermore, as the DCs were derived from different donors for **Figures 2**, **3A–F**, the *trans*-inhibitory activity of GPI-X5 appears to be conserved.

### GPI-scFv X5 expression in human primary CD4^+^ T cells blocks DC-mediated infection in trans

**Figure 4:**
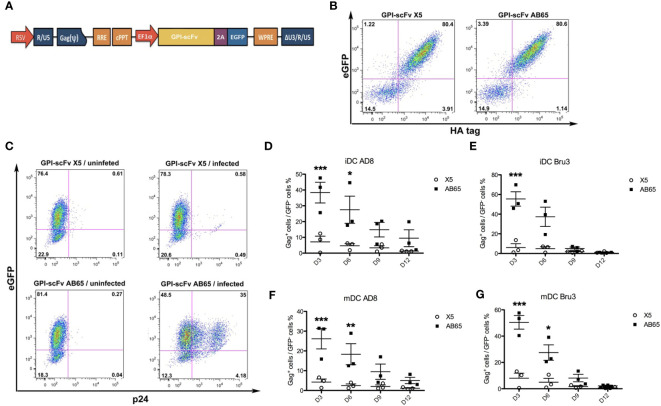
*GPI-scFv X5-transduced human primary CD4^+^ T cells are resistant to trans-infection of iDC- or mDC-captured HIV-1.* **(A)** Schematic diagram of lentiviral transfer vector pRRL-GPI-HA-scFv X5 or AB65-2A-GFP. RRE: rev response element; cPPT: central polypurine tract; EF1α: elongating factor 1α promoter; GFP: green fluorescent protein. **(B)** Expression of GFP and GPI-HA-scFv X5 (left panel) or AB65 (right panel) in human primary CD4^+^ T cells transduced with recombinant lentiviral vectors containing pRRL-GPI-HA-scFv X5 or AB65-2A-GFP, respectively, after a single round transduction. **(C)** A representative gating of p24^+^/GFP^+^ double positive cells in human primary CD4^+^ T cells transduced with recombinant lentiviral vectors containing pRRL-GPI-scFv X5 or AB65 *trans*-infected with or without iDCs containing captured HIV-1 AD8 at 2 days post co-culture. Human CD4^+^ T cells transduced with pRRL-GPI-scFv X5, but without iDCs-HIV-1 AD8 (upper-left panel). Human CD4^+^ T cells transduced with pRRL-GPI-scFv AB65, but without *trans*-infection with iDC-HIV-1 AD8 (lower left panel). Human CD4^+^ T cells transduced with pRRL-GPI-scFv X5 and cocultured with iDC- HIV-1 AD8 (upper right panel). Human CD4^+^ T cells transduced with pRRL-GPI-scFv AB65 and cocultured with iDC-HIV-1 AD8 (lower right panel). D to **(G)** Summary of percentages of Gag p24^+^/GFP^+^ cells at 3, 6, 9 and 12 days post co-culture from experiments using cells derived from three different human donors. **(D)** CD4^+^ T cells transduced with pRRL-GPI-HA-scFv X5 or AB65 and co-cultured with iDCs-HIV-1 AD8; **(E)** CD4^+^ T cells transduced with pRRL-GPI-scFv X5 or AB65 and co-cultured with iDCs-HIV-1 Bru-3; **(F)** CD4^+^ T cells transduced with pRRL-GPI-scFv X5 or AB65 and co-cultured with mDCs-HIV-1 AD8; **(G)** CD4^+^ T cells transduced with pRRL-GPI-scFv X5 or AB65 and co-cultured with mDCs-HIV-1 Bru-3. *stands for P<0.05; **stands for P<0.01; ***stands for P<0.001.

Having demonstrated that GPI-scFv X5 in transduced CD4^+^ CEMss cell lines effectively blocked HIV-1 infection *in trans* from iDC or mDC, we next evaluated whether GPI-scFv X5 transduced human primary CD4^+^ T cells would be protected from DC-mediated HIV-1 infection. To facilitate monitoring transduced human primary CD4^+^ T cells, we inserted genes encoding GPI-HA-scFv X5 or AB65 into the pRRL-2A-eGFP vector, which expresses an enhanced green fluorescent protein (eGFP) as a marker. The resulting pRRL-GPI-HA-scFv X5- or AB65- 2A-eGFP transfer vectors (**Figure 4A**) were used to produce recombinant pseudotypes for transduction of human primary CD4^+^ T cells. **Figure 4B** shows that after a single round of transduction, over 80% of human primary CD4^+^ T cells became GFP^+^/GPI-HA-scFv^+^ double positive cells.

To determine if GPI-scFv X5 could effectively block *trans*-infection of autologous primary CD4^+^ T cells, iDCs or mDCs with captured HIV-1 were co-cultured with GPI-scFv X5 or AB65-transduced human primary CD4^+^ T cells. After co-culturing, HIV-1 infection was measured by intracellular HIV-1 Gag p24 staining. **Figure 4C** shows the gating of p24^+^/GFP^+^ double positive cells in GPI-scFv X5 or AB65-transduced human primary CD4^+^ T cells co-cultured with or without iDCs with captured HIV-1 AD8. **Figures 4D**–**G** summarize the percentage of Gag p24^+^/GFP^+^ cells at 3, 6, 9 and 12 days post co-culture of iDCs or mDCs with captured HIV-1 AD8 or Bru-3 and GPI-scFv X5 or AB65-transduced human primary CD4^+^ T cells from three individual donors and independent experiments. Co-culture of iDCs containing HIV-1 AD8 and GPI-scFv AB65-transduced human primary CD4^+^ T cells resulted in significant increases in the percentages of p24^+^/GFP^+^ cells with an average 35.6% at the peak (3 days) post co-culture. In contrast, co-culture of iDCs-HIV-1 AD8 and GPI-scFv X5-transduced human primary CD4^+^ T cells resulted in little or no p24^+^/GFP^+^ cells throughout the experiments (**Figure 4D**). Similarly, co-culture of iDCs-HIV-1 Bru-3 and GPI-scFv AB65-transduced human primary CD4^+^ T cells resulted in significant increase in the percentages of p24^+^/GFP^+^ cells with the average 51.7% at the peak (3 days) post co-culture. In contrast, co-culture of iDCs-HIV-1 Bru-3 and GPI-scFv X5-transduced human primary CD4^+^ T cells resulted in little or no HIV-1 p24^+^/GFP^+^ cells throughout the experiments (**Figure 4E**). Similar results were also obtained when co-cultures were carried out between HIV-1 mDCs with captured HIV-1 AD8 or Bru-3 and GPI-scFv X5 or AB65-transduced human primary CD4^+^ T cells (**Figures 4F, G**). Thus, in transduced human primary CD4^+^ T cells, GPI-scFv X5 significantly blocks *trans*-infection of iDC- or mDC-captured HIV-1, further supporting the findings in **Figures 2**, **3**.

### Cell contact dependent inhibition of HIV-1 replication in iDCs by GPI-scFv X5 expressing CD4^+^ T cells

**Figure 5:**
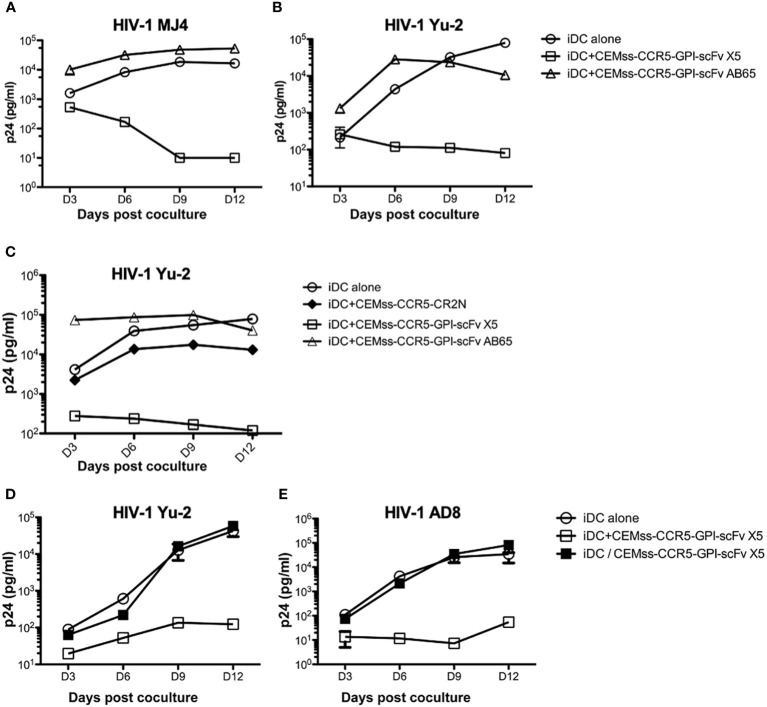
*Trans-blockage of R5-tropic HIV-1 in iDCs by GPI-scFv X5.* **(A)** HIV-1 Gag p24 in the culture supernatants collected at days 3, 6, 9 and 12 post co-culture between GPI-scFv X5 or AB65-transduced CEMss-CCR5 cells and iDCs with captured HIV-1 MJ4 or iDCs with HIV-1 MJ4 alone. **(B)** HIV-1 Gag p24 in the culture supernatants collected at days 3, 6, 9 and 12 post co-culture between GPI-scFv X5 or AB65-transduced CEMss-CCR5 cells and iDCs with captured HIV-1 Yu-2 or iDCs with HIV-1 Yu-2 alone. **(C)** HIV-1 Gag p24 in the culture supernatants collected at days 3, 6, 9 and 12 post co-culture between CEMss-CCR5-GPI-scFv X5 or AB65 or CEMss-CCR5-CR2N cells and iDCs with captured HIV-1 Yu-2 or iDCs with HIV-1 Yu-2 alone. D and **(E)** HIV-1 Gag p24 in the culture supernatants collected at days 3, 6, 9 and 12 post co-culture between CEMss-CCR5-GPI-scFv X5 cells and iDCs with captured HIV-1 Yu-2 **(D)** or AD8 **(E)** in a regular plate (open squares) versus a transwell plate (closed squares) or iDCs with HIV-1 Yu-2 **(D)** or AD8 **(E)** alone in a regular plate. Infections were performed in triplicate. The average values ± standard deviation (SD) are shown.

The co-culture experiments in **Figure 3A** suggested that expression of GPI-scFv X5 on T-cells may inhibit replication of R5-tropic HIV-1 AD8 in iDCs. To test whether replication of other HIV-1 strains in iDCs could be inhibited by GPI-scFv X5 expressing CD4+ T cells, we infected iDCs with two other R5-tropic HIV-1 clones, Mj4 and Yu-2. The iDCs were then co-cultured with or without CEMss-CCR5-GPI-scFv X5 or AB65 transduced cells. **Figures 5A, B** show that co-culture of iDCs containing either HIV-1 Mj4 or Yu-2 and CEMss-CCR5-GPI-scFv AB65 control resulted in high levels of HIV-1 production. Both viruses also replicated in the iDCs alone. In contrast, co-culturing iDCs infected with either HIV-1 Mj4 or Yu-2 and CEMss-CCR5-GPI-scFv X5 resulted in decreasing HIV-1 production. This experiment has been conducted two times with similar results. Thus, intercellular interactions may enable GPI-scFv X5 when expressed on CEMss-CCR5 cells to block replication of R5-tropic HIV-1 in neighboring iDCs.

To test whether restriction of R5-tropic HIV-1 in the iDC-T cell co-cultures is due to blocking of cell*-*cell transmission to CD4^+^ T cells expressing GPI-scFv X5, we co-cultured iDCs-HIV-1 Yu-2 with or without CEMss-CCR5-GPI-scFv X5 or AB65 or CEMss-CCR5-CR2N cells. In CEMss-CCR5-CR2N cells, the *ccr5* gene was disrupted using CRISPR/Cas9 system. Consequently, CEMss-CCR5-CR2N cells are rendered completely resistant to CCR5-tropic HIV-1 Yu-2 infection (41). Consistent with the experiment in **Figure 5B**, co-culture of iDCs-HIV-1 Yu-2 and CEMss-CCR5-GPI-scFv AB65 resulted in significantly higher levels of HIV-1 while co-culture of iDCs-HIV-1 Yu-2 with CEMss-CCR5-GPI-scFv X5 resulted in significant suppression of HIV-1 production than iDCs with virus alone (**Figure 5C**). Interestingly, co-culture of iDCs-HIV-1 Yu-2 and CEMss-CCR5-CR2N cells resulted in a delayed increase in virus production like iDCs with virus alone. While there is a small difference between iDC alone and iDC co-culture with CEMss-CCR5-CR2N, this was attributed to an over-growth of HIV-1 resistant CEMss-CCR5-CR2N cells, which reduced viability of the cells in the culture. These data indicate that CEMss-CCR5-CR2N cells resist *trans*-infection by iDC-captured HIV-1 Yu-2. However, without GPI-scFv X5 modification, these cells do not block viral replication in iDCs. Moreover, they suggest that the suppression of R5-tropic HIV-1 infection of iDCs observed in the co-cultures of iDCs-R5-tropic HIV-1 and GPI-scFv X5-expressing CEMss-CCR5 cells is not due to inhibition of *trans*-infection of the T cells by GPI-scFv X5.

To test whether the intercellular inhibition of R5-tropic HIV-1 replication in iDCs by GPI-scFv X5 transduced CD4^+^ T cells requires cell-cell contact, iDCs infected with CCR5 tropic HIV-1 AD8 or Yu-2 were co-cultured with or without CEMss-CCR5-GPI-scFv X5 separated by a trans-well barrier. **Figures 5D, E** show that co-culture of HIV-1 AD8 or Yu-2 infected iDCs and CEMss-CCR5-GPI-scFv X5 cells yields significantly lower levels of HIV-1 Gag p24 in the culture supernatants than co-culture of iDCs with only the viruses. Interestingly, separation of the iDCs and CEMss-CCR5-GPI-scFv X5 cells by a trans-well membrane abrogated the inhibitory effect, indicating that the intercellular inhibition of R5-tropic HIV-1 replication in iDCs by GPI-scFv X5 expressing CEMss-CCR5 cells requires close contact with iDCs. Taken together, these results suggest that expression of GPI-scFv X5 in neighboring T cells has the capacity to block HIV-1 replication *in trans* in iDCs.

### GPI-scFv X5-transduced T cells do not protect neighboring untransduced CD4^+^ T cells

**Figure 6:**
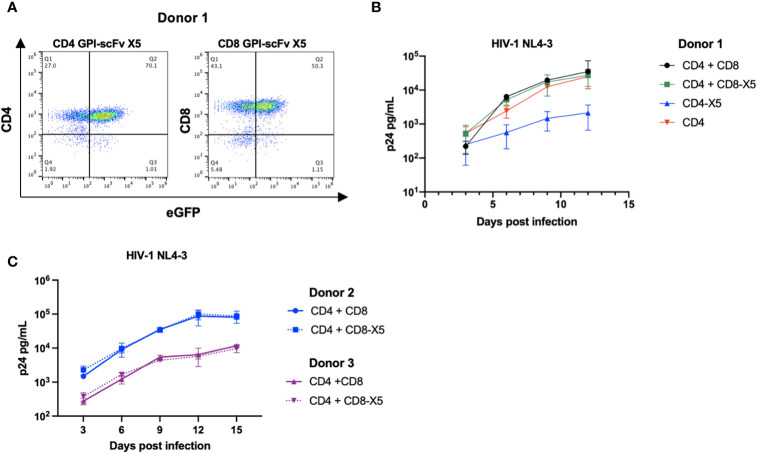
*GPI-scFv X5 protects CD4 T cells in cis but not in trans when displayed on CD8 T cells*. **(A)** Expression of CD4^+^ (left panel) or CD8^+^ (right panel) and GFP in human primary CD4^+^ T cells and CD8^+^ T cells transduced with recombinant lentiviral vectors containing pRRL-GPI-scFv X5-2A-GFP, respectively, after a single round transduction. **(B)** HIV-1 Gag p24 in the culture supernatants collected at days 3, 6, 9, and 12 post culture of CD4^+^, GPI-scFv X5 CD4^+^ or co-cultured with CD8^+^, or GPI-scFv X5 CD8^+^ infected with HIV-1 NL4-3. **(C)** Two additional donors, Donor 2 (blue) and Donor 3 (purple), were evaluated for GPI-scFv X5 modified CD8 T cell mediated trans protection of CD4 T cells. HIV-1 Gag p24 in the culture supernatants collected at days 3, 6, 9, 12, and 15 post co-culture of CD4^+^ with unmodified CD8^+^ (solid line) or GPI-scFv X5 CD8^+^ (dotted line) infected with HIV-1 NL4-3. Infections were performed in triplicate. The average values ± standard deviation (SD) are shown.

Having shown that expression of GPI-scFv X5 on CEMss *trans*-restricts HIV-1 in iDCs, we next evaluated if GPI-scFv X5 expression on T cells would also block infection of neighboring untransduced CD4^+^ T cells. Human primary CD4^+^ T cells and CD8^+^ T cells were transduced with the pRRL-GPI-scFv X5-2A GFP vector as described above (**Figure 4A**). **Figure 6A** shows that after a single round of transduction, over 70% of CD4^+^ T cells and 50% of CD8^+^ T cells became GFP^+^ positive.

Human primary CD4^+^ T cells or primary GPI-scFv X5 transduced CD4^+^ T cells were cultured alone or co-cultured with autologous human primary CD8^+^ T cells or primary GPI-scFv X5 transduced CD8^+^ T cells, and then infected with HIV-1 NL4-3. Expression of GPI-scFv X5 on CD4^+^ T cells resulted in significant suppression of HIV-1 p24 production than CD4^+^ T cells that do not express GPI-scFv X5 (**Figure 6B**). In contrast, co-culture of CD4^+^ T cells with CD8^+^ T cells or GPI-scFv X5 transduced CD8^+^ T cells resulted in a similar level of HIV-1 production, suggesting that expression of GPI-scFv X5 on CD8^+^ T cells does not enhance control of HIV-1 in cocultures with CD4^+^ T cells (**Figure 6B**). Similar results were observed in additional experiments using human primary CD4^+^ and CD8^+^ T cells derived from different donors (**Figure 6C**).

### GPI-anchored GFP migrates from transduced CD4^+^ T cells to iDCs

**Figure 7:**
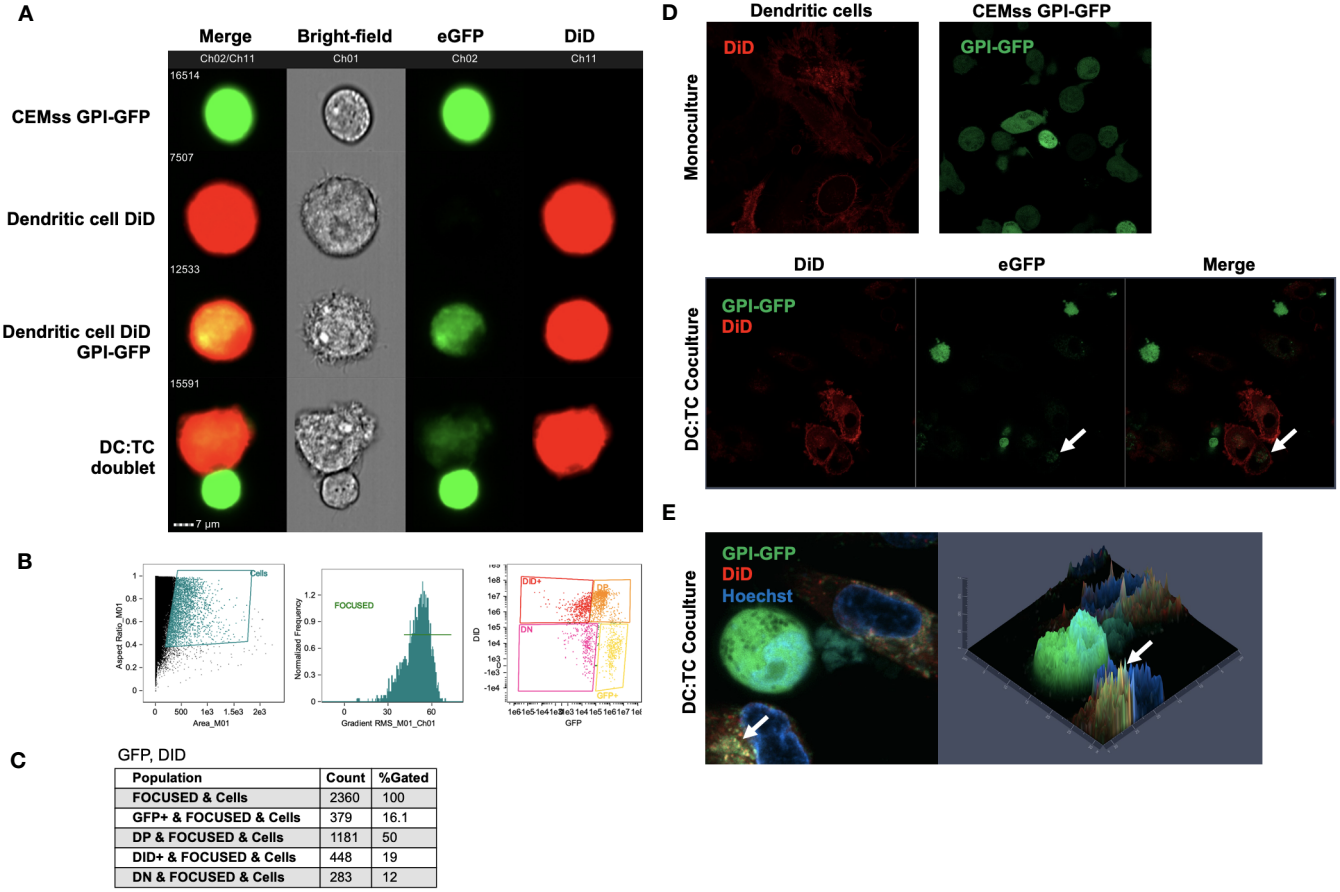
*Migration of GPI-GFP from GPI-GFP transduced CEMss cells to iDCs.* **(A)** Representative images by Amnis ImageStream Mark-II of single cell GPI-GFP CEMss cells, DiD dyed iDCs, GPI-GFP transfer to DiD dyed iDCs and cell-cell conjugates between DiD dyed iDCs and GPI-GFP CEMss cells. T cells are shown in Ch02 expressing GPI anchored GFP. DCs dyed with DiD are shown in Ch11. **(B)** Gating strategy for analysis of DC : TC cocultures. Cells were analyzed by aspect ratio vs area to identify single cells. RMS feature of Ch1 (Bright-field) were then used to plot gradient by normal frequency to select for focused events. Cells were finally selected for positivity in both Ch02 and Ch11 looking for expression of eGFP and DiD, respectively. **(C)** Population count of total cells sorted and counts of expression for GFP positive, double positive (DP), DiD positive, or double negative (DN) populations and relative percent. **(D)** AiryScan Confocal microscopy of monoculture DiD dyed DCs, CEMss GPI-GFP, or DC : TC coculture. The top left panel shows a representative image of DC monocultures stained with DiD dye. The top right panel shows CEMss cells that express GPI anchored GFP. The bottom three panels show DC : TC coculture at a 1:1 ratio. GPI-GFP was detected on DiD dyed dendritic cells when imaged after 2 hours of coculture as indicated by white arrows. **(E)** Left panel shows representative image by AiryScan confocal microscopy of DC : TC cocultures after 2 hours. DCs were pre-stained with DiD and cocultured with GPI-GFP CEMss cells. Cocultures were then stained with Hoechst nuclear blue dye. White arrows indicate detection of GPI-GFP on DCs. Right panel shows fluorescence signal intensity plot. Yellow punctae indicate GPI-GFP co-localization with DiD.

To determine potential mechanisms of inhibition of HIV replication in iDCs by GPI-scFv X5 expressing CD4^+^ T cells, we first examined if recombinant GPI-anchored proteins can be transferred from transduced CEMss cells to iDCs. To evaluate transfer of GPI-anchored proteins, CEMss cells were transduced with a GPI-green fluorescent protein (GFP). iDCs were marked using a fluorescent DiD membrane dye and incubated with CEMss GPI-GFP for 2 hours. After co-culture, the movement of GPI-GFP were evaluated by Amnis ImageStream live-cell imaging. **Figure 7A** shows that after co-culture, GPI-GFP is significantly enriched on DiD dyed iDCs. Further, iDC and CEMss GPI-GFP cell-cell synapse can be seen where GPI-GFP is enriched on iDCs suggesting that transfer of GPI-GFP can occur from GPI-GFP transduced CEMss to iDCs after 2 hours of co-culture and may occur through cell-cell synapse engagement (**Figure 7A**). Cell populations were gated and analyzed by DiD and eGFP expression showing 50% of cells were double positive, indicating that the transfer of GPI anchored GFP occurs in half of cells when cocultured (**Figures 7B, C**). Next, we wanted to further confirm colocalization of GPI-GFP with the membrane dye DiD. Transfer of GPI-GFP from transduced CEMss cells to iDCs were evaluated after 2 hours of co-culture by Airyscan confocal microscopy. A similar pattern of enrichment of GPI-GFP in DiD dyed iDCs was also observed (**Figures 7D, E**). GPI-GFP is enriched on iDCs and co-localization of GPI-GFP and DiD dye on iDCs forming yellow punctae on iDCs were observed (**Figure 7E**). Taken together, GFP when anchored to GPI and expressed on CEMss cells by a single round of transduction migrates from CEMss transduced cells to iDCs after 2 hours of co-culture.

### GPI-scFv X5 migrates and is displayed on the surface of iDCs from GPI-scFv transduced CEMss cells

While we demonstrated that GPI-GFP could migrate from GPI-GFP transduced CEMss cells to iDCs, we next determined if scFvs anchored by GPI attachment were also transferred to iDCs. Further, it is possible that GPI-GFP may be taken up by iDCs internally and sequestered in vesicles but not displayed on the surface of the cell membrane. To evaluate this, we used CEMss cells transduced with the recombinant lentiviral vector pRRL-GPI-HA-scFv X5-2A-GFP. Cells transduced by pRRL-GPI-HA-scFv X5-2A-GFP result in internal GFP expression, marking transduced cells, while HA tagged GPI-scFvs are anchored to the outer leaflet of the plasma membrane. As previously shown, after a single round of transduction, cells are double positive for GPI-scFv and GFP (**Figures 4A, B**).

**Figure 8:**
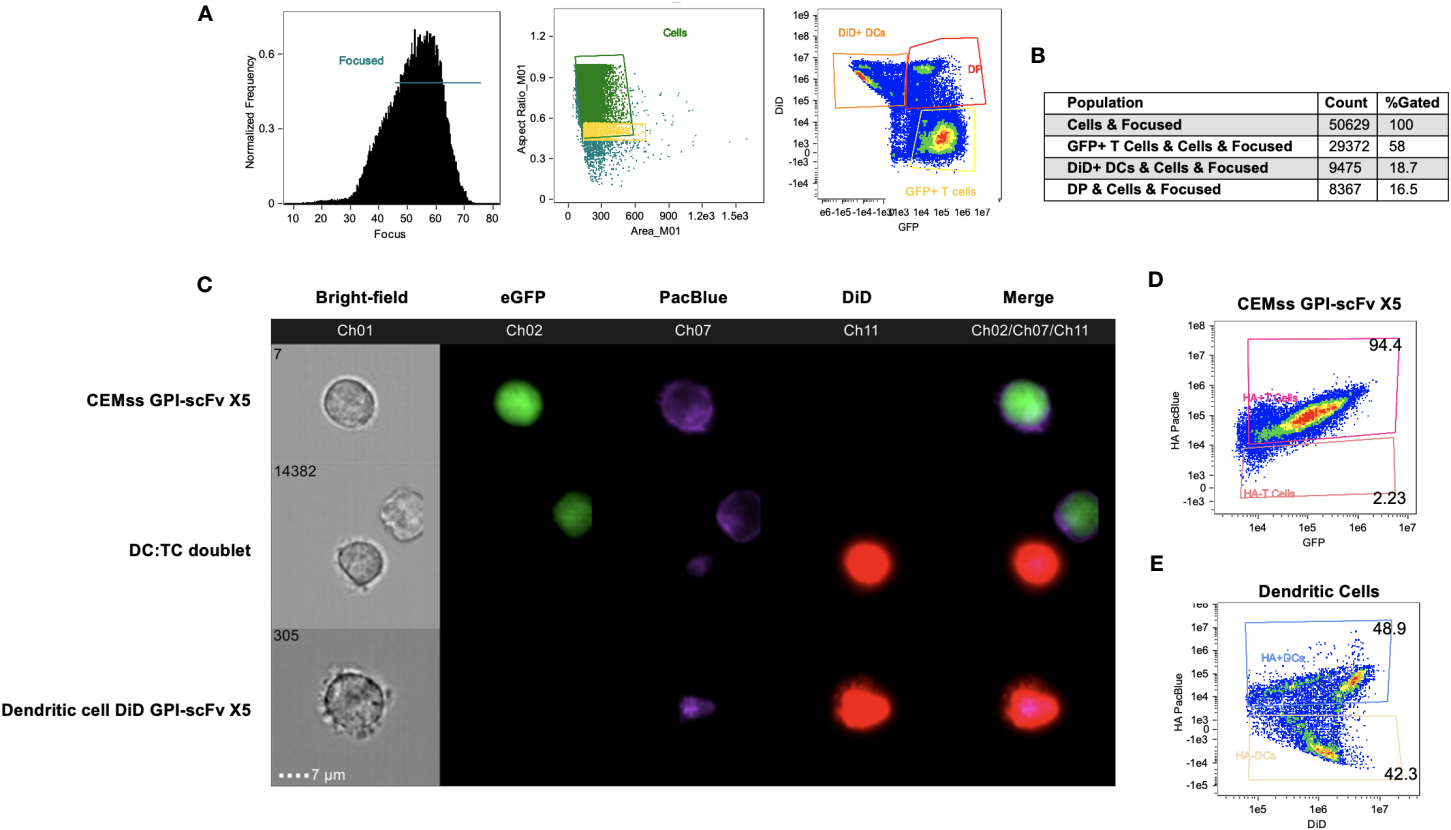
*GPI-scFv X5 is transferred from CEMss cells to dendritic cells*. GPI-scFv X5 CEMss cells were cocultured with DiD dyed DCs for 2 hours and analyzed by Amnis ImageStream Mark-II. **(A)** Gating strategy for analysis of DC : TC cocultures. RMS feature of Ch1 (Bright-field) were used to plot gradient by normal frequency to select for focused events. Cells were then analyzed by aspect ratio vs area to identify single cells and likely doublets. Cells were finally selected for positivity in both Ch02 and Ch11 looking for expression of eGFP and DiD, respectively. **(B)** Population count of total cells sorted and counts of expression for GFP positive, DiD positive, or double positive (DP) populations and relative percent. **(C)** Representative images of CEMss GPI-scFv X5 co-expressing GFP and GPI-HA-scFv X5 (top panel), DC : TC doublets show anti-HA PacBlue tagged GPI-HA-scFv X5 detected on DCs after DC : TC coculture (middle panel), and DiD dyed DC positive for GPI-HA-scFv X5 (bottom panel). **(D)** Coexpression of HA PacBlue and GFP on GPI-scFv X5 CEMss cells are 94.4% double positive. **(E)** DiD dyed DCs analyzed for coexpression of DiD and HA PacBlue after 2 hours of coculture with GPI-HA-scFv X5 CEMss reveals two distinct population of DiD+ HA PacBlue+ (48.9%) and DiD+ HA PacBlue- (42.3%).

GPI-scFv X5 CEMss cells were cultured with DiD dyed iDCs for 2 hours and analyzed by Amnis ImageStream Mark-II. Surface expression of HA within the GPI-scFv X5 construct was then antibody-labeled with a PacBlue-conjugated anti-HA tag antibody. In focused cells, single versus doublet cells were gated by aspect ratio and subsequently analyzed for DiD and GFP expression (**Figure 8A**). Over 50,000 events were imaged with 16% of focused cells double positive for both GFP and DiD (**Figure 8B**). In **Figure 8C**, GPI-scFv X5 labeled with PacBlue-conjugated anti-HA are seen abundantly displayed on the surface of transduced CEMss GPI-scFv X5 cells that are marked by internal GFP expression. In DC : TC doublets, GPI-scFv X5 labeled by anti-HA PacBlue is seen enriched on the surfaces of iDCs dyed with DiD, suggesting that GPI-scFv X5 is transferred from transduced CEMss cells to iDCs after 2 hours of co-culture. Importantly, anti-HA PacBlue labeled GPI-scFv X5 is detected on the surface of DiD labeled iDCs, but not GFP, which is internally expressed in transduced CEMss cells, indicating that scFvs when anchored to GPI can be transferred from T cells to iDCs in co-culture (**Figure 8C**). When analyzing CEMss GPI-scFv X5 transduced cells, over 94% of cells are double positive for both GFP and PacBlue labeled HA tag after a single round of transduction (**Figure 8C**). Additionally, almost 50% of untransduced iDCs received GPI-scFv X5 from CEMss cells as detected by surface expression of HA tagged GPI-scFv X5 after just 2 hours of co-culture with CEMss GPI-scFv X5 transduced cells, (**Figure 8E**). Furthermore, GPI-scFv X5 CEMss cells when cocultured with primary CD4^+^ T cells were not found to pass GPI-scFv X5 from transduced CEMss cells to untransduced primary CD4^+^ T cells (**Figures S4A–C**). That is, in TC : TC co-cultures, GPI-X5 does not transfer from transduced CD4^+^ T cells to untransduced CD4^+^ T cells. Additionally, when DCs were added to TC : TC co-culture, transfer of GPI-scFv X5 from transduced CEMss cells to iDCs and subsequently from iDCs to naïve human primary CD4 T cells was not observed (**Figures S5A, B**).

Taken together, these data show that GPI-scFv X5 on transduced CEMss cells migrate to iDCs and are displayed on the outer membrane of iDCs after 2 hours of co-culture. This suggests that protection of iDCs *in trans* mediated by contact with CEMss GPI-scFv X5 cells may be due to transfer of GPI-scFv X5 from transduced CEMss to iDCs.

## Discussion

HIV-1 transmission occurs by both cell-free and cell-cell infection with cell-cell mechanisms found to be more efficient in spreading virus and less susceptible to inhibition by neutralizing antibodies and entry inhibitors than cell-free virus infection (13–15, 17–26). Furthermore, increasing evidence indicate that cell-cell transmission of HIV-1 may play an important role in the establishment of systemic infection as well as in virus spread within lymphoid tissues *in vivo* (16, 42). For example, Mooruka et al. showed that in a BLT humanized mouse model HIV-1-infected T cells in lymph nodes through virological synapses. Blocking the egress of T cells from lymph nodes into efferent lymph vessels at the onset of HIV-1 infection limited HIV-1 dissemination and reduced plasma viremia (16, 43). Sewald et al. showed that in secondary lymph tissues, HIV-1 and murine leukemia virus (MLV) are first captured by sinus-lining macrophages via CD169 that recognizes gangliosides. Virus-captured by macrophages then *trans*-infect B-1 cells. Infected B-1 cells then migrate into the lymph node to spread infection through virological synapses (42). Thus, development of an effective way to block cell-cell transmission of HIV-1 could have potential advantages for HIV-1 prevention and therapy.

In the present study, we demonstrated that after co-culturing iDCs or mDCs with captured HIV-1 and GPI-scFv-transduced CD4^+^ cell lines as well as human primary CD4^+^ T cells, the anti-HIV-1 GPI-scFv X5 inhibitor almost completely blocks *trans*-infection of the target cells (**Figures 2**–**4**). Importantly, CD4 is a lipid raft-associated protein and richly present in the contact zone (7, 35, 36). The epitope recognized by antibody X5 resides within a conserved region (amino acid residues 417 to 434) of the Env gp120 core, which is nearby the CD4 and co-receptor binding sites (44). We envision that when CD4 binds the native envelope spike on virions, GPI-scFv X5 residing in lipid rafts may quickly and efficiently engage the CD4-induced transiently exposed epitope in Env. This may block the conformational transition of Env at a pre-fusion intermediate step, and inhibit viral entry.

One surprising finding in this study is that GPI-scFv X5 not only effectively blocks HIV-1 cell-cell infection of CD4^+^ cell lines or human primary CD4^+^ T cells *in trans* from either iDC or mDC-captured HIV-1, but it also blocks HIV-1 infection of the iDCs (**Figures 3**, **5**). The *trans*-inhibitory activity requires cell-cell contact with GPI-scfv X5 transduced CD4^+^ T cells. Inhibition was not observed when iDCs with captured HIV-1 were co-cultured with a R5-tropic HIV-1-resistant cell line, CEMss-CCR5-CR2N cells (**Figure 5**), indicating that the *trans*-infection neutralization of R5-tropic HIV-1 replication in iDCs is not due to the infection resistant phenotype of GPI-scFv X5-transduced CEMss-CCR5 cells. Additionally, GPI-scFv X5 expression on human primary CD8^+^ T cells does not enhance neutralization of HIV-1 in CD4^+^ T cells when co-cultured (**Figure 6**). Thus, the trans-inhibitory effect of GPI-scFv X5 may depend on stable synapses that occur between CD4^+^ T cells and iDCs, but not between T cells.

Using live cell microscopy and imaging flow cytometry, we demonstrated transfer of engineered GPI-anchored proteins from transduced T cells to iDCs using a GPI-GFP construct and GPI-scFv X5. The migration of GPI-GFP from transduced CEMss T cells to iDCs was observed when cells were co-cultured (**Figure 7**). Additionally, we found that GPI-scFv X5 migrates from CEMss transduced cells to iDCs in co-culture (**Figure 8**). These data provide further support that recombinant GPI-anchored proteins can be transferred from transduced T cells to untransduced iDCs. Importantly, we also showed that GPI-scFv X5 is displayed on the surface of iDCs where it would be available to neutralize infection. By contrast, GPI-scFv X5 was not observed to be transferred from transduced T cells to non-transduced T cells. Consequently, GPI-scFv X5 transduced T cells do not neutralize HIV-1 replication in non-transduced CD4^+^ T cells in coculture. Together, these data further support transfer of anti-HIV GPI-anchored scFv as a mechanism of inhibition of viral infection of iDCs.

Cell-cell transfer of GPI-anchored proteins has been shown *in vivo*, such as transfer of complement restriction factors CD55 and CD59 from erythrocytes to the vascular epithelium and from male genital tract epithelium to spermatozoa (45). The latter serves a physiological function during sperm maturation, sperm storage and fertilization (46). Thus, if cell-cell transfer of GPI-scFv X5 also occurs *in vivo*, this should have important implications for the utility of GPI-scFv inhibitor-based therapy against HIV-1.

Finally, anti-HIV GPI-anchored scFv inhibitors with such remarkable breadth of neutralization activity against both cell-free and cell-cell transmissions of HIV-1 should have potential either alone or in the combination with other anti-HIV-1 gene constructs, such as GPI-HCDR3 PG16 or GPI-C34 fusion inhibitor, to be developed into anti-viral agents for HIV-1 prevention and therapy (40, 47). For example, GPI-scFv-X5 and GPI-HCDR3 PG16, due to their different specificities, could be co-delivered into hematopoietic progenitor cells or human primary CD4^+^ T cells of HIV-1 patients *ex vivo* through lentiviral vector transduction. The GPI-anchored inhibitor modified hematopoietic progenitor cells or human primary CD4^+^ T cells could then be transfused to the patients as described by DiGiusto et al. and Tebas et al., respectively (48, 49). Alternatively, GPI-scFv X5 could be used to modify and protect HIV-specific T cells to improve their persistence and activity (50–53). HIV specific T cells are a reservoir that contributes to persistence and immune dysfunction (54). Protecting them from new rounds of infection may improve immune function and reduce the viral reservoir. However, many hurdles, such as efficient engraftment, self-renewal, and linage cell differentiation, sustainable transgene expression, avoidance of potential insertion mutagenesis, have to be worked out in HIV-1 infection humanized mouse models or SHIV infection macaque models before the clinical efficacy of GPI-scFv X5 or GPI-HCDR3 PG16-transduced hematopoietic progenitor cells or human primary CD4^+^ T cells can be tested in human patients. Taken together, we show that GPI-scFv X5 may be a promising approach for HIV-1 prevention and therapy by restricting HIV-1 *cis-* and *trans-*infection mechanisms of CD4^+^ cells.

## Materials and methods

### Cell lines

The packaging cell line 293FT was purchased from Invitrogen Life Technologies and maintained in complete DMEM medium [i.e. high glucose DMEM supplemented with 10% FBS, 2 mM L-glutamine, 1 mM sodium pyruvate, penicillin (100 U/ml), streptomycin (100 μg/ml)] plus G418 (500 μg/ml) (Invitrogen Life Technologies). CEMss-CCR5 cells and stably transduced TZM.bl-GPI-scFv X5, TZM.bl-GPI-scFv AB65, CEMss-CCR5-GPI-scFv X5, CEMss-CCR5-GPI-scFv AB65 and CEMss-CCR5-CR2N were generated before and maintained in complete DMEM (41, 55).

### TZM.bl cells

The TZM.bl cell line was obtained from J. Kappes and X. Wu via the NIH AIDS Research and Reference Reagent Program (ARRRP; Germantown, MD). TZM.bl cells are a well described reporter cell line for their utility in HIV-1 studies. Briefly, TZM.bl cells are derived from HeLa cells that have been transduced to express HIV-1 receptors and co-receptors CD4, CCR5, and CXCR4, respectively (34). The cell line was additionally modified by lentiviral vectors to express an HIV-1 promoter controlling *E. coli* β-galactosidase and firefly luciferase reporters (56, 57). Thus, infection of TZM.bl cells by HIV-1 triggers expression of reporter constructs by HIV-1 Tat mediated mechanisms. Quantitation of luciferase by luminescence intensity and reported as Relative Light Units (RLU) were conducted utilizing the BrightGlo luciferase activity kit according to the manufacturer’s instructions (Promega).

### Human monocyte-derived iDCs and mDCs and primary CD4 T cells

Human peripheral blood mononuclear cells (PBMCs) of healthy donors were purchased from the Gulf Coast Regional Blood Center in Houston, Texas, Shanghai Blood Center or from the Blood Bank of the Shanghai Hospital, Shanghai, China. The iDCs were prepared as previously described (37). Briefly, human monocytes were isolated from buffy coats using a Ficoll gradient and a subsequent CD14 selection step using the MACS system according to manufacturer’s instructions (Miltenyi Biotec). Purified monocytes were differentiated into iDCs in the presence of IL-4 (50 ng/ml) and GM-CSF (50 ng/ml) (R&D System). On day 6, iDC phenotype was confirmed by flow cytometry using antibodies against CD3, CD11c, CD14, CD83, CD86 and CD209 (CD-SIGN) (see below).

To generate mDCs, iDCs were further cultured in the presence of LPS (100 ng/ml) for another 2 days. mDC phenotype was confirmed by flow cytometry using antibodies against CD83, CD86, CD209 and HLA-DR (see below).

Human primary CD4^+^ T cells were enriched from above CD14^+^ monocyte-depleted PBMCs by negative selecting magnetic beads according to the manufacturer’s instructions (Thermo Fisher Scientific) and resuspended in the complete RPMI 1640 medium (i.e. RPMI 1640 medium supplemented with 15% FBS, 2mM L-glutamine, 1 mM sodium pyruvate, penicillin [100 U/ml], and streptomycin [100 μg/ml]) supplemented with human rIL-2 (100 IU/ml, R&D System) before being activated and transduced with recombinant lentiviruses (see below).

### Gene constructs

Fusion genes encoding the GPI-scFv X5 or AB65 were amplified by PCR using pRRL-GPI-scFv X5 or AB65 as templates with a pair of primers (Forward: 5’-CCATGGGCTTGCTGCTGACTGGCAGCGGCGCCACCAACTTCA-3’, Reverse: 5’-AGTCGCCGTGAACGTTCTTTT-3’). GPI-scFv X5 or AB65 genes were transferred into pRRLsin-18.PPT.EF1a.GFP.Wpre (41). The resulting lentiviral transfer constructs were designated pRRL-GPI-scFv X5 or AB65-2A-GFP.

### Generation of recombinant lentiviral viruses

Recombinant lentiviral viruses were generated as described previously (37). Briefly, 4 X 10^6^ 293FT cells were seeded onto a P-100 dish in 10 ml complete DMEM. After culturing overnight, cells were co-transfected with 20 μg transfer construct pRRL-GPI-scFv X5 or AB65-2A-GFP, 10 μg packaging construct encoding HIV-1 Gag/Pol (pLP1), 7.5 μg plasmids encoding the vesicular stomatitis virus G protein envelope (pLP/VSV-G), and 7.5 μg HIV-1 Rev protein (pLP2) (Invitrogen) using a calcium phosphate precipitation method. Sixteen hours later, culture supernatants were removed and replaced with fresh complete DMEM plus 1 mM sodium butyrate (Sigma). After eight hours, supernatants were again removed and replaced with fresh DMEM plus 4% FBS. After another 20 hours, the culture supernatants were harvested and concentrated by ultracentrifugation. The recombinant lentivirus pellets were resuspended in a small volume of DMEM and stored in aliquots in a -80°C freezer. Recombinant lentivirus titers were determined as previously described (37).

### Transducing GPI-scFv X5 or AB65 into human primary CD4 T cells

To transduce human primary CD4^+^ T cells, human primary CD4^+^ T cells were enriched from CD14^+^ monocyte-depleted PBMCs by negative selecting magnetic beads (see above). 2.5 × 10^5^ human CD4 T cells per well were activated by mixing with anti-CD3/CD28 antibody-coated beads (Thermo Fisher Scientific) at 1:1 ratio in 500 µl complete RPMI 1640 medium supplemented with human rIL-2 (100 IU/ml) in 48 well plates. After 24 hours, 5 x 10^6^ TCID recombinant lentiviral viruses containing pRRL-GPI-scFv X5 or AB65-2A-GFP in complete RPMI 1640 supplemented with human rIL-2 (100 IU/ml) and 8 µg/ml polybrene were added into cell suspension at final volume of 750 µl. The plates were centrifuged at 1,500g and 37°C for 2 hours to facilitate transduction. After overnight incubation at 37°C, 500 µl supernatant was removed and 750 µl fresh complete RPMI 1640 medium supplemented with human rIL-2 (100 IU/ml) were added into cells. The anti-CD3/CD28 antibody-coated beads in human CD4^+^ T cells were removed by DynaMag magnet after 4 days activation. Transduced human CD4^+^ T cells were resuspended in 2 ml complete RPMI 1640 supplemented with human rIL-2 (100 IU/ml) and cultured in 24-well plates for additional 2 days. Transduction efficiency was estimated by GFP and GPI-scFv X5 or AB65 expression using FACS analysis. Untransduced human CD4^+^ T cells were used as a recipient cells in the *trans*-infection study (see below).

### HIV-1 viruses and pseudotypes

HIV-1 molecular clones AD8, Yu2, Mj4 (CCR5 tropic) and Bru-3 (CXCR4 tropic) were produced by transfecting proviral plasmids pAD8 (58), pYu2 (59), pMj4 (60) and pBru-3 (61) into 293 FT cells using a calcium phosphate precipitation as described before (37). The 50% tissue culture infection dose (TCID_50_) was determined by serial titration of viruses in TZM.bl cells (37).

To generate HIV-1 pseudotypes, 4 X 10^6^ 293FT packaging cells were co-transfected with 10 μg of an HIV-1-luciferase transfer vector and 1 μg of a DNA plasmid encoding one of several HIV-1 envelopes Bru-3, AD8, Yu2, JRFL, consensus B, consensus C, CNE3 and CNE11 using a calcium phosphate precipitation method (62). AD8, Yu2 and JRFL are derived from R5-tropic subtype B viruses (58, 63–66). CNE3 is derived from an R5-tropic CRF01_AE recombinant (66). CNE11 is derived from an R5-tropic subtype B’ virus. The pseudotype-containing supernatants were harvested and stored in aliquots at -80°C. Relative Light Units (RLU) of HIV-1 or 10A1 pseudotypes were determined as previously described (62).

### FACS analysis

To determine the phenotype of iDCs, monocyte-derived iDCs were incubated with PE-conjugated anti-CD83, CD86, DC-SIGN, or HLA-DR, and FITC-conjugated anti-CD14 and APC-conjugated anti-CD3, CD11c antibodies (BD BioSciences) for 45 min on ice. To determine the phenotype of mDCs, monocyte-derived mDCs were incubated with PE-conjugated anti-CD83, CD86, DC-SIGN or HLA-DR antibodies for 45 min on ice. Cells then were washed twice with FACS buffer (PBS containing 1% BSA and 0.02% NaN_3_) and fixed with 1% formaldehyde in 0.5 ml of FACS buffer. FACS analysis was performed on a FACScan LSR II (Becton Dickinson, Mountain View, CA). FACS data were analyzed with FlowJO 7.6.1.

To determine transgene expression, 3 X 10^5^ mock-, pRRL-GPI-scFv X5 or AB65-2A-GFP-transduced human primary CD4 T cells were stained with rabbit anti-HA tag antibody at 4^0^C for 40 min. Cells were washed twice with FACS buffer and stained with Alexa633 conjugated goat anti-rabbit IgG antibody on ice for 30 min. Cells then were washed twice with FACS buffer (PBS containing 1% BSA and 0.02% NaN_3_) and fixed with 1% formaldehyde in 0.5 ml of FACS buffer. FACS analysis was performed on a FACScan LSR II (Becton Dickinson, Mountain View, CA). FACS data were analyzed with FlowJO 7.6.1.

Intracellular HIV-1 Gag p24 detection was performed as described before (17). Briefly, at various indicated time intervals post co-culture between HIV-1-captured iDCs or mDCs and pRRL-GPI-scFv X5 or AB65-2A-GFP-transduced human primary CD4 T cells, small portion of cell mixture was harvested, washed with 1 ml FACS buffer, fixed and permeabilized by Cytofix/Cytoperm kit (Becton Dickinson). Cells were re-suspended in 50ul Perm/Wash buffer with 0.8 µl PE-conjugated anti-Gag p24 antibody (KC57, Beckman Coulter) and incubated for 30 min on ice. After washing twice with 1ml Perm/Wash buffer, the cells were re-suspended in 400 µl FACS buffer and analyzed by FACS Fortessa (Becton Dickinson) using software FlowJo and GraphPad Prism.

### Trans-infection of DC-captured HIV-1 to target cells

To examine DC-mediated HIV-1 infection of GPI-scFv transduced TZM.bl cells, human iDCs or mDCs were incubated with HIV-1 AD8 and Bru3 at MOI 5 for 2 hours in triplicate cultures. Cells were then washed 3 times with PBS to remove cell-free virions and seeded in triplicate in a 96 wells flat-bottom plate (2×10^4^ per well) along with or without mock-, GPI-scFv X5- or AB65-transduced TZM.bl cells (1×10^4^ per well). The cell-cell transmission of HIV-1, as measured by RLU in cell lysates, was determined after 48 hours by a BrightGlo Luciferase assay according to the manufacturer’s instructions (Promega).

To examine *trans*-infection of GPI-scFv transduced CEMss-CCR5 cells by DC-captured HIV-1, human iDCs or mDCs were incubated with HIV-1 AD8, Bru-Yu2, Mj4 and Bru3 at MOI 5 for 2 hours in triplicate cultures. Cells were then washed 3 times with PBS to remove cell-free virions and seeded in triplicate in a 24 wells flat-bottom plate (1×10^5^ per well) along with or without GPI-scFv X5 or AB65-transduced CEMss-CCR5 or CEMss-CCR5-CR2N cells (2×10^5^ per well). Cell mixtures were cultured in a total volume of 2 ml for 12 days. Every 3 days, 1.2 ml culture supernatants were collected and replaced with fresh complete RPMI 1640 medium. HIV-1 Gag p24 in the supernatant was measured by ELISA according to the manufacturer’s instructions (Zepto Metrix Co.).

In transwell experiments, 1 x 10^6^ iDCs were incubated with HIV-1 AD8 or Bru-Yu2 at MOI of 5 at 37°C for 2 hours in triplicate cultures and washed extensively to remove cell-free virions. 2 x 10^5^ CEMss-CCR5-GPI-scFv X5 or AB65 cells were seeded in 800 µl complete RPMI 1640 in the upper chamber of 24-well transwell plates (Corning) and 1 X 10^5^ HIV-1-captured iDCs in 1ml RPMI1640 were added at the bottom chamber. Cells were incubated for 12 days. Every 3 days, 500 μl cells in upper layer were replaced by fresh complete RPMI 1640, while 670 µl supernatant in bottom layer were collected and replenished with fresh complete RPMI 1640 medium. The amount of HIV-1 Gag p24 in the culture supernatants was measured by ELISA according to the manufacturer’s instructions.

To test *trans*-infection of transduced human primary CD4^+^ T cells by DC-captured HIV-1, iDCs or mDCs were incubated with HIV-1 AD8 and Bru3 at MOI 5 for 2 hours in triplicate cultures. Cells were then washed 3 times with PBS to remove free viruses and seeded in triplicate in a 96 wells flat-bottom plate (2×10^5^ per well) along with or without pRRL-GPI-scFv X5 or AB65-2A-GFP-transduced human primary CD4^+^ T cells (1×10^5^ per well). Cell mixtures were co-cultured in a total volume of 200 µl for 12 days. at 6 hours, 1, 2, 3, 6, 9 and 12 days, small portions of cell suspension were harvested and replaced with fresh complete RPMI 1640 medium and human rIL-2 (100 IU/ml). Intracellular HIV-1 Gag p24 was analyzed by FACS (see above).

### Trans-infection with HIV-1 pseudotyped virions

3×10^5^ iDCs or mDCs were incubated with HIV-luc pseudotyped with either HIV-1 Env proteins or the 10A1 control in triplicate cultures. The amount of viral particles added to the DCs was equivalent to 2 ng HIV-1 Gag p24 at 37°C. After a 2 hour incubation, cells were washed extensively. 2×10^5^ CEMss-CCR5-GPI-scFv X5 or AB65 cells were co-cultured with 1×10^5^ iDCs or mDCs harboring captured virions, or iDCs or mDCs without virions, at 37°C for 48 hours in 1ml complete RPMI 1640 in 48wells plates. The cocultures were collected for assessing luciferase activity using BrightGlo luciferase activity kit according to the manufacturer’s instructions (Promega).

### Infection of human primary CD4 and CD8 T cell co-cultures

To evaluate if expression of GPI-scFv X5 on CD8 T cells enhances restriction of HIV-1 *in trans* of CD4 T cells, primary cells were isolated and transduced as previously described. Briefly, CD4 T cells were isolated from healthy human PBMCs and activated by PHA (5ug/ml) stimulation and expanded for one week. CD4 and CD8 T cells were transduced using lentiviral vectors as described above. Transduced or untransduced CD4 cells were plated in triplicate on a 96 well U-bottom plate (VWR). 2.5×10^5^ cells CD4 and CD8 were co-cultured at a 1:1 ratio and suspended in 100 µl of RPMI media supplemented with IL-2 (100 IU/ml). Cell cultures in triplicate were infected for three hours with HIV-1 NL4-3 at an MOI of 0.01. Plates were spun down at 1200 rpm for 5 minutes and cells were washed two times with PBS. Co-cultures were collected and resuspended in 1 ml of RPMI supplemented with IL-2 (100 IU/mL) in 24 well plates. Supernatants were collected at three day intervals post infection and replenished with complete media. Experiments were independently performed with cells from three different donors.

### Live-cell imaging

To evaluate migration of GPI-GFP to iDCs, 1×10^6^ iDCs were suspended in 250 µl of FluoroBrite DMEM (ThermoFisher) with 5 µl Vybrant DiD-Cell Labeling Solution (ThermoFisher) for 10 minutes at 37°C. iDCs were then washed three times and incubated in 500 µl FluoroBrite DMEM at a 1:1 ratio with CEMss-GPI GFP cells at 37°C for two hours in a 5mL polystyrene tube (Falcon). In iDC co-culture experiments with CEMss-GPI-HA-scFv X5 cells, co-cultures were stained with PacBlue conjugated anti-HA tag antibody (BioLegend) at 37°C for 40 min. Cells were washed twice with FACS buffer. Unfixed co-cultures were then resuspended in 50 µl FluoroBrite DMEM in micro-centrifuge tubes (Avantor VWR). FACS analysis and imaging was performed on a Amnis ImageStream Mk II Imaging Flow Cytometer (Luminex, Austin, TX). ImageStream data were analyzed with IDEAS 6.3.

To obtain confocal images of GPI-GFP transfer to iDCs, 5×10^5^ iDCs were resuspended in 300 µl of complete DMEM and seeded in 8 well chamber slides (IBIDI) 24 hours before imaging. Media was removed and iDCs were washed once with PBS and replaced with FluoroBrite DMEM. 1.5 µl Vybrant DiD-Cell Labeling Solution (ThermoFisher) was added for 10 minutes at 37°C. Cells were then washed twice with PBS and 5×10^5^ CEMss-GPI GFP cells in 300 µl of FluoroBrite DMEM were co-cultured with iDCs. Images were obtained after two hours by a LSM 980 with AiryScan 2 Confocal Microscope (Zeiss). Images were analyzed with ZEN 3.5.

### Statistical analysis

The data in **Figures 2**, **4** were analyzed by two-way ANOVA and post-test Bonferroni comparison using GraphPad software. The data in **Figures 5**, **6** were compared by unpaired *t*-test using the same software. If 0.01≤p ≤ 0.05, it was labeled as *, while if 0.001≤p ≤ 0.01, it was labeled as **; if p ≤ 0.001, it was labeled as ***.

## Data availability statement

The original contributions presented in the study are included in the article/**Supplementary material**. Further inquiries can be directed to the corresponding authors.

## Ethics statement

Ethical approval was not required for the studies on humans in accordance with the local legislation and institutional requirements because only commercially available established cell lines were used.

## Author contributions

KT, WW, PZ and JK conceived the project and designed the experiments. KT, WW, CY, SS, HH, LL, MW, and AM performed experiments and analyzed the data. KT, WW, JK, and PZ wrote the manuscript. The co-first authors, KT and WW, contributed equally and both earned the right list their name first in their C.V. All authors contributed to the article and approved the submitted version.
