# Supplementary material for: Engineered CD4 T cells expressing a membrane anchored viral inhibitor restrict HIV-1 through cis and trans mechanisms
Source: Front Immunol. 2023 Sep 14;14:1167965. doi: 10.3389/fimmu.2023.1167965 (PMC10538569; doi:10.3389/fimmu.2023.1167965)
Supplement: Supplementary file 1 [file DataSheet_1.docx]

**Supplementary Figures: Engineered CD4 T cells expressing a membrane anchored viral inhibitor restricts HIV-1 through *cis* and *trans* mechanisms**


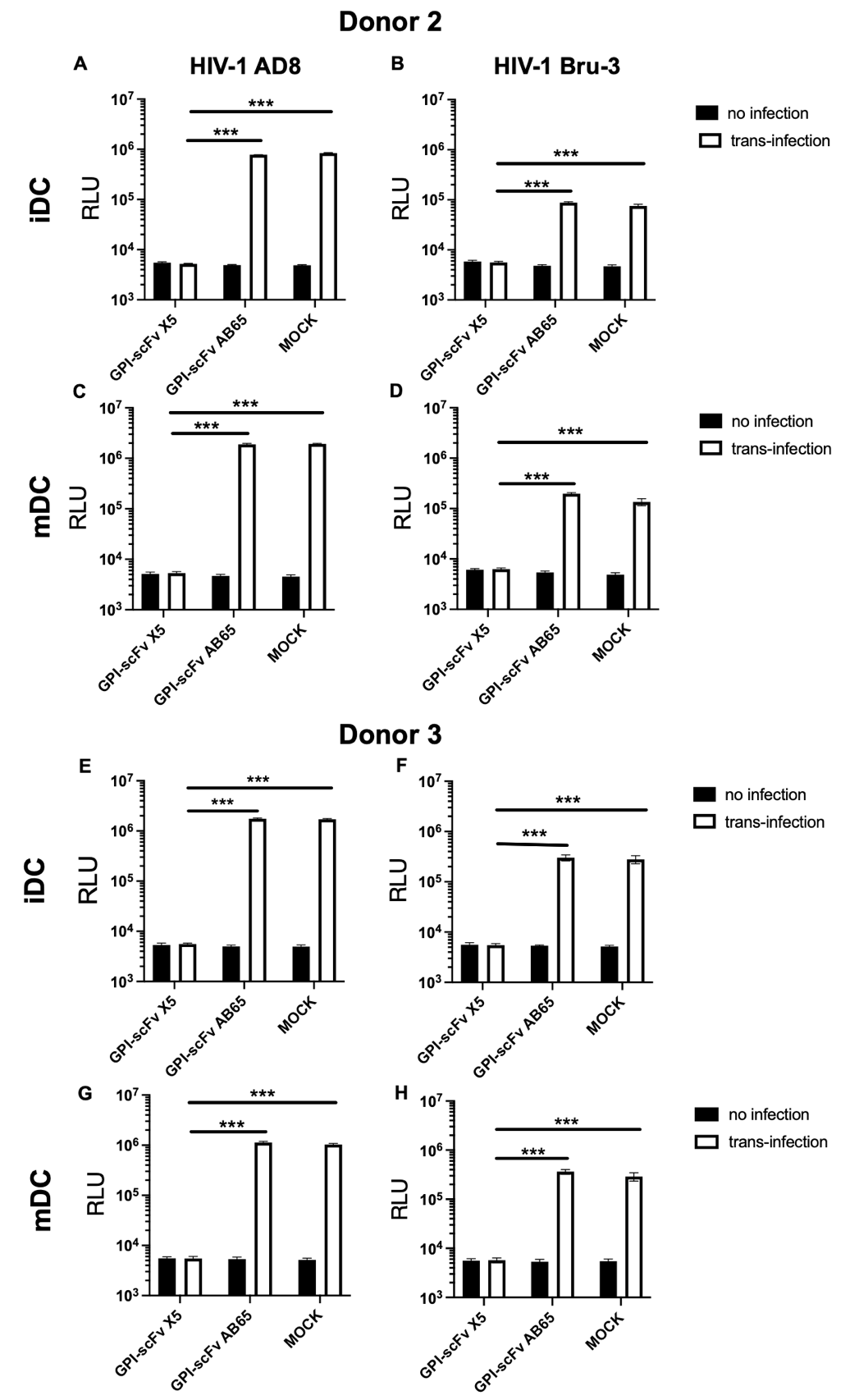


**Figure S1.** *GPI-scFv X5-transduced TZM.bl cells are resistant to iDC- or mDC-mediated trans-infection of HIV-1 in cocultures using DCs derived from two additional different donors.* Donor 2 A to D and Donor 3 E to H. A and E. RLU in mock-, GPI-scFv X5 or AB65-transduced TZM.bl cells with or without *trans*-infection of iDC-captured HIV-1 AD8. *** stands for *P* values = or < 0.001. B and F. RLU in mock-, GPI-scFv X5 or AB65-transduced TZM.bl cells with or without *trans*-infection of iDC-captured HIV-1 Bru-3. *** stands for *P* values = or < 0.001. C and G. RLU in mock-, GPI-scFv X5 or AB65-transduced TZM.bl cells with or without *trans*-infection of mDC-captured HIV-1 AD8. Infections were performed in triplicate. The average values ± standard deviation (SD) are shown. *** stands for *P* values = or < 0.001. D and H. RLU in mock-, GPI-scFv X5 or AB65-transduced TZM.bl cells with or without *trans*-infection of mDC-captured HIV-1 Bru-3. *** stands for *P* values = or < 0.001.


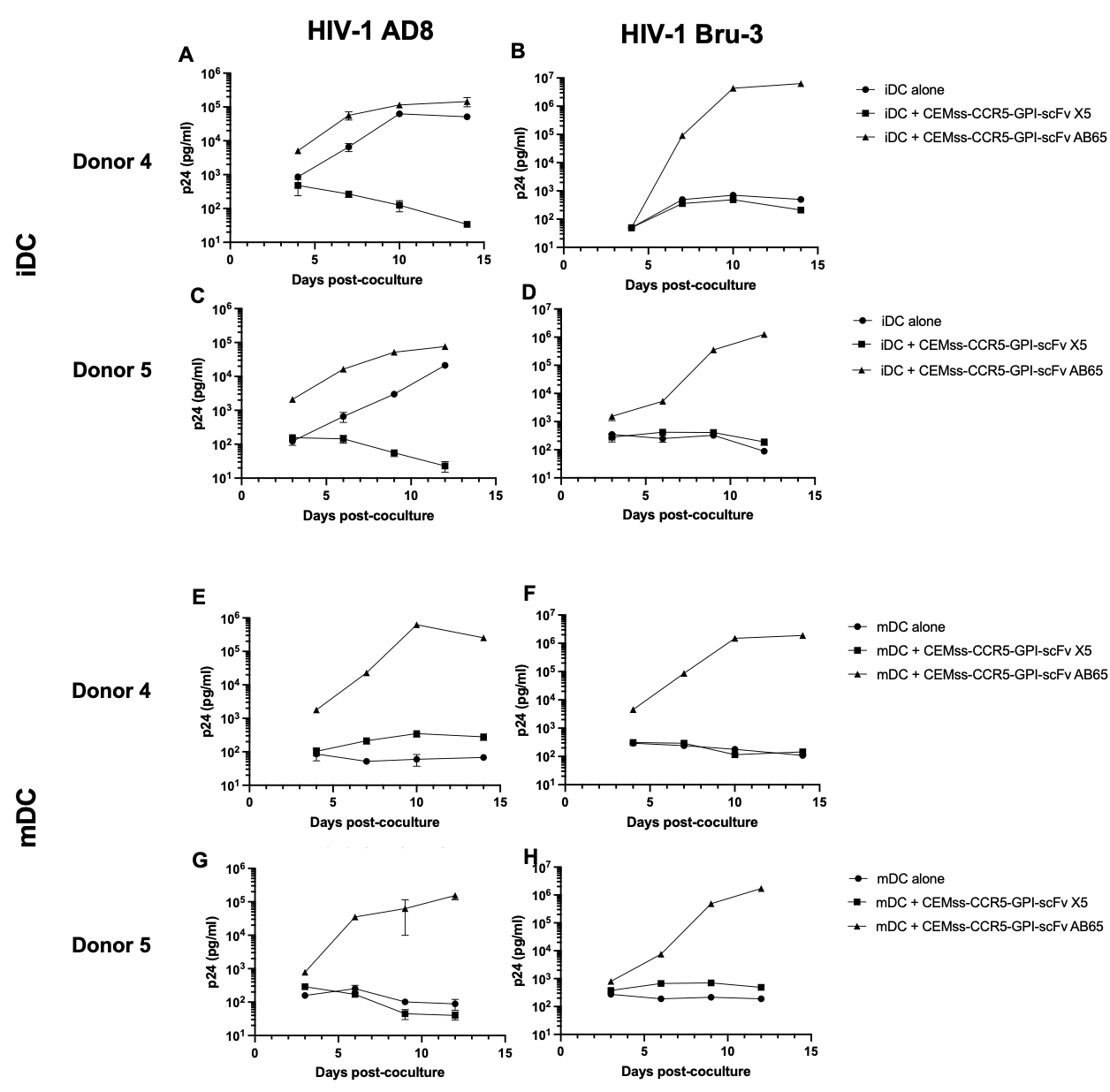


**Figure S2.** *GPI-scFv X5-transduced CEMss-CCR5 cells block HIV-1 replication in cocultures with either iDCs or mDCs derived from two additional blood donors.* Donor 4 A, B, E and F. Donor 5 C, D, G and H. A and E. HIV-1 gag p24 in the culture supernatants collected at days 3, 6, 9 and 12 post co-culture between mock-, GPI-scFv X5 or AB65-transduced CEMss-CCR5 cells and HIV-1 AD8-captured iDCs. B and F. HIV-1 gag p24 in the culture supernatants collected at days 3, 6, 9 and 12 post co-culture between mock-, GPI-scFv X5 or AB65-transduced CEMss-CCR5 cells and HIV-1 Bru-3-captured iDCs. C and G. HIV-1 gag p24 in the culture supernatants collected at days 3, 6, 9 and 12 post co-culture between mock-, GPI-scFv X5 or AB65-transduced CEMss-CCR5 cells and HIV-1 AD8-captured mDCs. D and H. HIV-1 gag p24 in the culture supernatants collected at days 3, 6, 9 and 12 post co-culture between mock-, GPI-scFv X5 or AB65-transduced CEMss-CCR5 cells and HIV-1 Bru-3-captured mDCs.


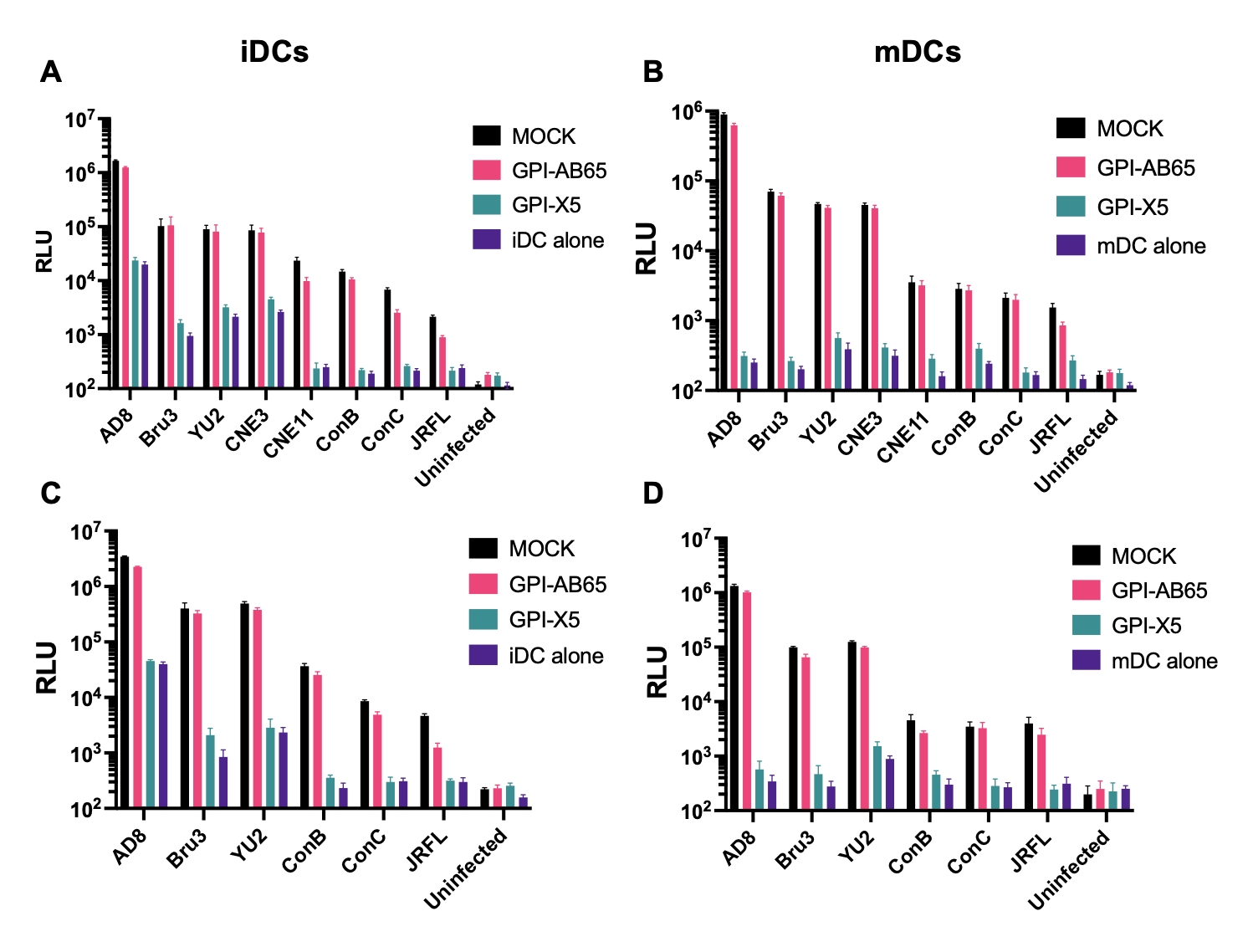


**Figure S3*.*** *Panel of iDC- or mDC-captured HIV-luc pseudotyped with different Env proteins and GPI-scFv X5-transduced CEMss-CCR5 cells show resistance to trans-infection of HIV-1 using DCs derived from additional blood donors.* Different donor derived DCs were used for independent experiments in panels A and B, C, and D. A*.* RLU detected in mock-, GPI-scFv X5 or AB65-transduced CEMss-CCR5 cells *trans*-infected with or without HIV-1 Env pseudotyped AD8, Bru-3, Yu2, Con B, Con C, JRFL-captured iDCs at 48 hours post co-culture. (A) included additional CNE3 and CNE11-captured iDCs. RLU in HIV-1 pseudotyped AD8, Bru-3, Yu2, CNE3, CNE11, Con B, Con C or JRFL-captured iDCs alone were included for the comparison. B and D. RLU detected in mock-, GPI-scFv X5 or AB65-transduced CEMss-CCR5 cells *trans*-infected with or without HIV-1 pseudotyped AD8, Bru-3, Yu2, Con B, Con C or JRFL-captured mDCs at 48 hours post co-culture. (B) included additional CNE3 and CNE11-captured mDCs. RLU in HIV-1 pseudotyped AD8, Bru-3, Yu2, CNE3, CNE11, Con B, Con C or JRFL-captured mDCs alone were included for the comparison. Infections were performed in triplicate. The average values ± standard deviation (SD) are shown.


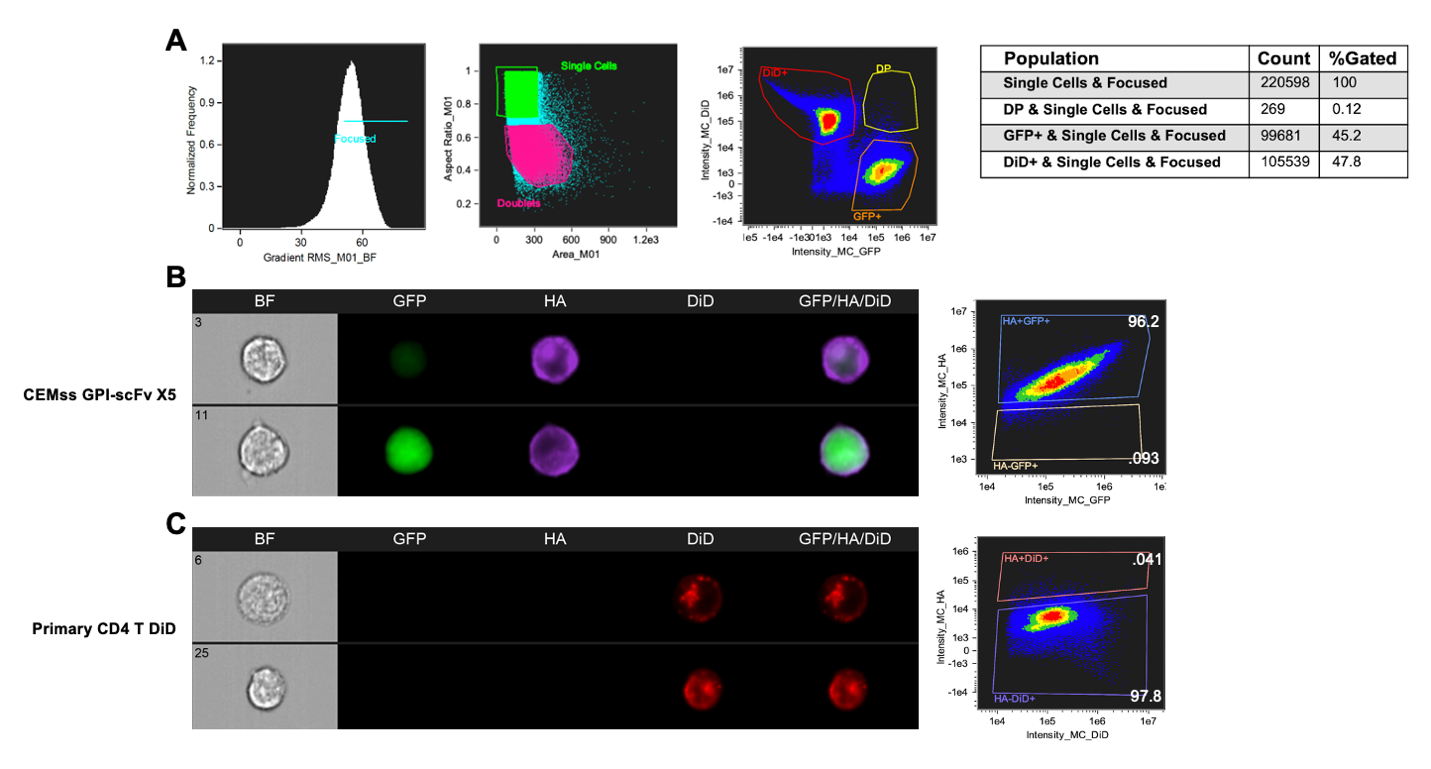


**Figure S4.** *GPI-scFv X5 is not transferred between CD4 T cells.* GPI-scFv X5 CEMss cells were cocultured with DiD dyed primary human CD4 T cells isolated from healthy donors at a 1:1 ratio for 2 hours and analyzed by Amnis ImageStream Mark-II. A. Gating strategy for analysis of TC:TC cocultures. RMS feature of Ch1 (Bright-field) were used to plot gradient by normal frequency to select for focused events. Cells were then analyzed by aspect ratio vs area to identify single cells and likely doublets. The left panels show cells gated for positivity in both Ch02 and Ch11 looking for expression of eGFP and DiD, respectively. The right table shows population count of total cells sorted and counts of expression for GFP positive, DiD positive, or double positive (DP) populations and relative percent. B. Representative images of CEMss GPI-scFv X5 co-expressing GFP and GPI-HA-scFv X5 (left panels). The right panel shows 96.2% of transduced CEMss expressing GFP and PacBlue conjugated anti-HA tag when gating by GFP and HA co-expression. C. Representative images of primary CD4 T cells dyed with DiD (left panels). The right panel shows that 97.8% of untransduced primary CD4 T cells were negative for PacBlue conjugated anti-HA tag when gating by GFP and HA co-expression.


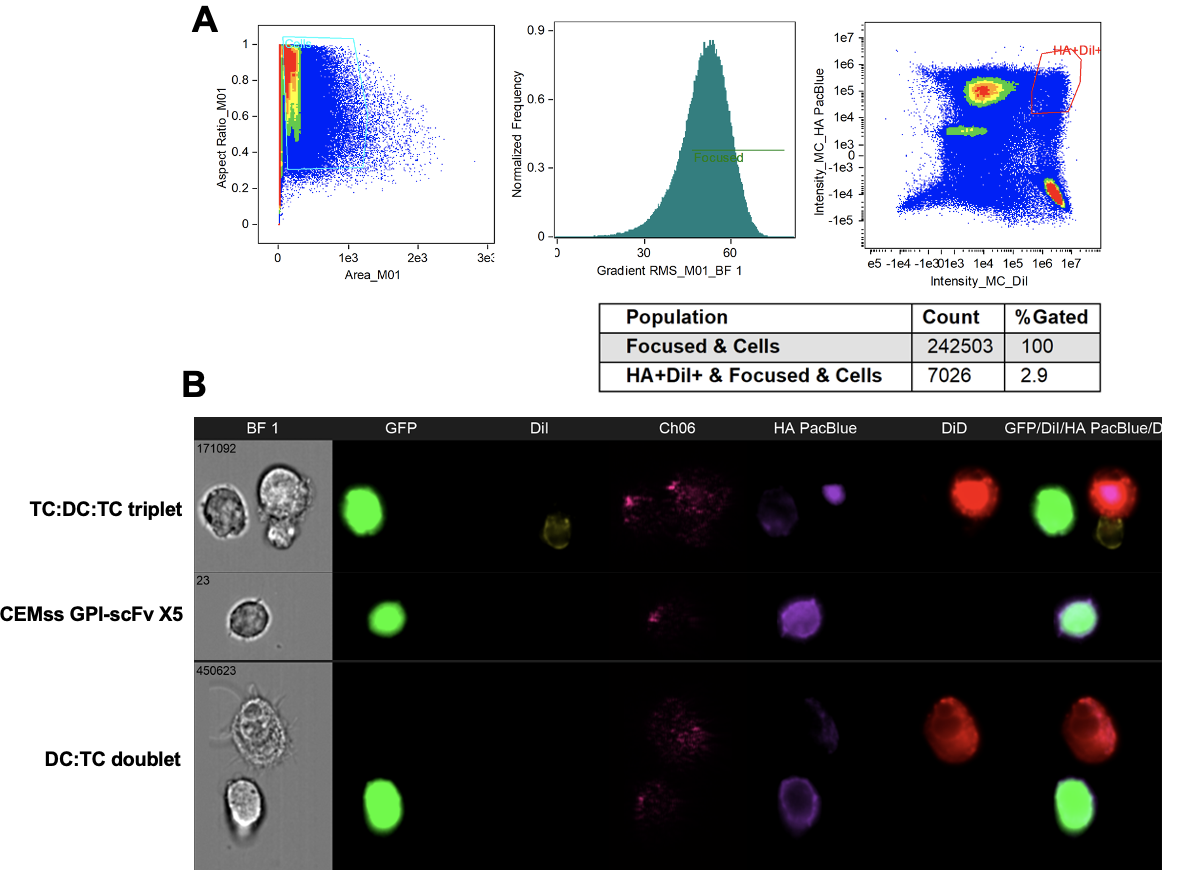


**Figure S5.** *GPI-scFv X5 is not transferred from transduced to naïve CD4 T cells in co-culture with Dendritic cells.* GPI-scFv X5 CEMss cells were cocultured with DiI dyed primary human CD4 T cells isolated from healthy donors and DiD dyed monocyte derived dendritic cells at a 1:1:2 ratio for 2 hours and analyzed by Amnis ImageStream Mark-II. A. Gating strategy for analysis of TC:TC:DC cocultures. Cells were analyzed by aspect ratio vs area to identify single cells and likely doublets. RMS feature of Ch1 (Bright-field) were then used to plot gradient by normal frequency to select for focused events. The top panels shows cells gated for expression of DiI and HA PacBlue. The bottom table shows population count of total cells sorted and counts of expression for focused cells analyzed and double positive populations for HA and DiI with relative percent gated. B. Representative images of DiI dyed human primary CD4 T cell with CEMss-GPI-scFv X5 cells coexpressing GFP and HA and DiD dyed dendritic cell with Bright-field (BF 1) and SSC (Ch06) (top panel). Representative image of CEMss GPI-scFv X5 co-expressing GFP and GPI-HA-scFv X5 (middle panel). Representative image of dendritic cells dyed with DiD with CEMss transduced T cells (bottom panel).
